# Systematic Review and Meta-analysis of the Additional Benefit of Pharmacological Thromboprophylaxis for Endovenous Varicose Vein Interventions

**DOI:** 10.1097/SLA.0000000000005709

**Published:** 2022-10-07

**Authors:** Benedict R. H. Turner, Matthew Machin, Sara Jasionowska, Safa Salim, Sarah Onida, Joseph Shalhoub, Alun H. Davies

**Affiliations:** Academic Section of Vascular Surgery, Department of Surgery and Cancer, Charing Cross Hospital, Imperial College London, London, UK

**Keywords:** anticoagulation, compression stockings, deep vein thrombosis, pulmonary embolism, radiofrequency ablation, thromboprophylaxis, ultra-sound guided foam sclerotherapy, varicose veins, venous thromboembolism

## Abstract

The primary objective of this systematic review and meta-analysis was to elucidate the rate of venous thromboembolism (VTE) after endovenous interventions for varicose veins in the presence of pharmacological and mechanical thromboprophylaxis versus mechanical thromboprophylaxis alone.

**Background:**

The VTE rate after endovenous procedures for varicose veins is higher than other day-case procedures and could be reduced with pharmacological thromboprophylaxis.

**Methods:**

The review followed Preferred Reporting Items for Systematic Reviews and Meta-Analysis guidelines with a registered protocol (PROSPERO: CRD42021274963). Studies of endovenous intervention for superficial venous incompetence reporting the predefined outcomes with at least 30 patients were eligible. Data were pooled with a fixed effects model.

**Results:**

There were 221 trials included in the review (47 randomized trial arms, 105 prospective cohort studies, and 69 retrospective studies). In randomized trial arms, the rate of deep venous thrombosis with additional pharmacological thromboprophylaxis was 0.52% (95% CI, 0.23%–1.19%) (9 studies, 1095 patients, 2 events) versus 2.26% (95% CI, 1.81%–2.82%) (38 studies, 6951 patients, 69 events) with mechanical thromboprophylaxis alone. The rate of pulmonary embolism in randomized trial arms with additional pharmacological thromboprophylaxis was 0.45% (95% CI, 0.09–2.35) (5 studies, 460 participants, 1 event) versus 0.23% (95% CI, 0.1%–0.52%) (28 studies, 4834 participants, 3 events) for mechanical measures alone. The rate of EHIT grade III to IV was 0.35% (95% CI, 0.09–1.40) versus 0.88% (95% CI, 0.28%–2.70%). There was 1 VTE-related mortality and 1 instance of major bleeding, with low rates of minor bleeding.

**Conclusions:**

There is a significant reduction in the rate of DVT with additional pharmacological thromboprophylaxis and routine prescription of anticoagulation after endovenous varicose vein intervention should be considered. VTE risk for individual study participants is heterogeneous and risk stratification in future randomized interventional studies is critical to establish the clinical effectiveness and safety of additional pharmacological thromboprophylaxis.

## BACKGROUND

The rate of venous thromboembolism (VTE) after endovenous intervention for superficial venous incompetence has been the topic of considerable debate,^1–3^ but remains higher than other day-case surgical procedures such as laparoscopic cholecystectomy, or major abdominal procedures such as colectomy.^4–6^ Though mortality after endovenous procedures remains a rarity, deaths have been reported in the media, secondary to pulmonary emboli (PE) from deep venous thromboses (DVT).^7,8^ Of those developing VTE, 30% will experience a recurrence within 10 years and 20% of patients with PE will die before diagnosis.^9^ Moreover, DVT carries a serious risk of morbidity through the development of postthrombotic syndrome up to 20% after isolated distal DVT (Turner BRH, Thapar A, Jasionowska S, Machin M, Gwozdz AM, Davies AHD. Systematic review and meta-analysis of the rate of post-thrombotic syndrome after isolated distal DVT. European Journal of Vascular and Endovascular Surgery [unpublished]).^10,11^

Thromboprophylaxis can effectively reduce VTE occurrence and is subdivided into mechanical measures, such as compression stockings or compression bandages, and pharmacological measures, such as anticoagulant medications. The most recent European Society of Vascular Surgery guidelines suggest an individualized VTE risk assessment for every patient undergoing varicose vein intervention.^12^ Although anticoagulants are commonly administered after varicose vein intervention,^13^ there is a lack of consensus as to the optimal pharmacological thromboprophylaxis strategy. This study aimed to pool previously published data to elicit the rate of DVT and PE in trial arms with mechanical and additional pharmacological thromboprophylaxis versus mechanical thromboprophylaxis alone.

## METHODS

The Preferred Reporting Items for Systematic Reviews and Meta-Analysis guidance was followed in conducting database searches.^14^ The study protocol was preregistered and is freely accessible (PROSPERO: CRD42021274963).

### Search Strategy

The MEDLINE, Embase, and Cochrane databases were accessed in December 2021, without search limitations imposed. Unpublished literature was screened by searching for trials on ClinicalTrials.gov, European Union Clinical Trials, and the International Standard Randomised Controlled Trial Number (ISRCTN). Searches used appropriate terms for varicose veins, VTE, and any endovenous varicose vein interventions (Figure, Supplemental Digital Content 1, http://links.lww.com/SLA/E261).

### Eligibility Criteria

Included articles had the following characteristics:

1. Studies or trial arms reporting the rate of DVT or PE
2. Patients with superficial venous reflux undergoing endovenous intervention to obliterate truncal reflux
3. Thirty patients or more

Excluded articles had the following characteristics:

1. Open surgery for venous reflux
2. No mechanical thromboprophylaxis
3. Full text not available in the English language, review articles, case reports, conference abstracts, or duplicate publications

### Article Screening

The articles were screened independently against the eligibility criteria by 2 reviewers (B.R.H.T. and S.J.); any discrepancies were mediated by a third reviewer (M.M.). The reference lists of all included articles were screened. The screening process was conducted on EndNote X20 with titles and abstracts screened before full text review.

### Data Extraction and Quality Assessment

Extraction of data was performed independently by 2 reviewers using a pre-defined template on Microsoft Excel. Quality assessment was also performed independently by two reviewers using the Cochrane Risk of Bias and Risk of Bias in Non-Randomised Studies of Interventions tools. Any discrepancies were mediated by a third reviewer (M.M.). If specific details regarding mechanical thromboprophylaxis were not specified, data were treated pragmatically and concordance with NICE and manufacturer guidelines, which mandate post-operative compression, was assumed. Studies that administered compression for less than the recommended 1-week period were included with further subgroup analysis planned.

### Statistical Analysis

Outcome data were pooled with a *metaprop* analysis in the program R using the meta package. Narrative synthesis was conducted where data were not suitable for meta-analysis. Where heterogeneity was suitably low, a fixed-effects model was used to pool data. A logit transformation was used normalize the data, in light of the skewed distribution expected from the low proportion of events.^15^

## RESULTS

In total, 221 trial arms and studies were included in the quantitative meta-analysis with a total of 476,266 participants (Figure, Supplemental Digital content 2, http://links.lww.com/SLA/E262, Preferred Reporting Items for Systematic Reviews and Meta-Analysis flow diagram of included studies). Studies consisted of 47 randomized trials, 105 prospective cohort studies, 67 retrospective cohort studies, and 2 case control studies (Table, Supplemental Digital Content 3, http://links.lww.com/SLA/E263 for a further summary of baseline study data). The indication for endovenous treatment in all studies was superficial venous incompetence, most commonly defined as >0.5 seconds in the standing position, with a range of clinical, aetiological, anatomical and pathological classification class 1 to 6 chronic venous insufficiency. The studies utilized a mixture of endovenous techniques including endovenous laser ablation, radiofrequency ablation, mechanochemical ablation, cyanoacrylate glue, and ultrasound-guided foam sclerotherapy. The majority of studies examined the great saphenous vein in isolation; studies treating reflux in the small saphenous vein (SSV) and anterior accessory saphenous vein were also included. The adjunctive use of mini-phlebectomies, avulsions, and/or sclerotherapy for the management of tributaries or perforators was ubiquitous, thus these data were not collected. The vast majority of studies excluded participants with a past history of VTE.

There were 43 study arms (13,839 participants) in which mechanical and additional pharmacological thromboprophylaxis were administered compared with 184 study arms (462,397 participants) with mechanical thromboprophylaxis alone. Of studies administering anticoagulants, 12 opted for a single prophylactic dose of low–molecular weight heparin (LMWH). Extended pharmacological thromboprophylaxis was defined as any continued administration of anticoagulation more than a single one-off dose and 29 studies adopted this with a further 2 studies using a combination of single-dose and extended pharmacological thromboprophylaxis. Extended regimes included 3 to 14 days of prophylactic dose LMWH, vitamin K antagonists, and direct oral anticoagulants. Overall, LMWH was the most commonly used anticoagulant agent.

Mechanical thromboprophylaxis was mostly commonly delivered with compression stockings, or compression bandaging in the case of venous leg ulceration. The reporting of the details of compression therapy was highly variable; where possible, class of compression, duration of use, and adherence with compression were collected.

### DVT

**FIGURE 1:**
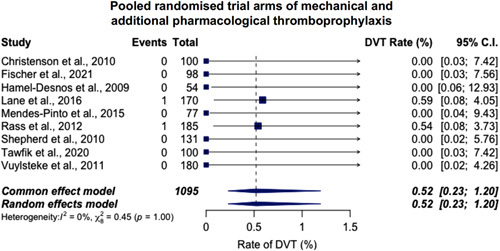
The pooled incidence of DVT with anticoagulation and mechanical thromboprophylaxis for endovenous varicose vein surgery in randomized trial arms. The rate of DVT is 0.52% (95% CI, 0.23%–1.19%) (9 studies, 1095 patients, 2 events).

**FIGURE 2:**
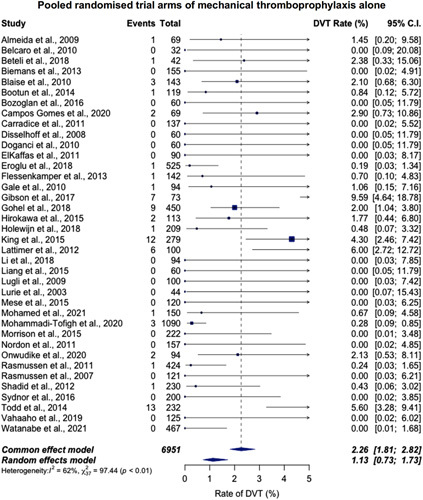
The pooled incidence of DVT with mechanical thromboprophylaxis alone for endovenous varicose vein surgery in randomized trial arms. The rate of DVT is 2.26% (95% CI, 1.81%–2.82%) (37 studies, 6951 patients, 69 events).

The rate of DVT was extracted in all 221 studies. For randomized trial arms, the rate of DVT in the mechanical and additional pharmacological thromboprophylaxis arms was 0.52% [95% confidence interval (CI) 0.23%–1.19%] (9 trial arms, 1095 patients, 2 events) (Fig. 1). For mechanical thromboprophylaxis alone, the rate of DVT was 2.26% (95% CI, 1.81%–2.82%) (38 trial arms, 6951 patients, 69 events) (Fig. 2). Heterogeneity, expressed via *I* ^2^, was 0% and 62%, respectively. Across all prospective study designs, the rate of DVT in the additional pharmacological thromboprophylaxis arms was 0.73% (95% CI, 0.52%–1.02%) (31 study arms, 6151 participants, 23 events, *I* ^2^=0%) versus 1.31% (95% CI, 1.16%–1.48%) (123 study arms, 36,418 participants, 214 events, *I* ^2^=60%) for mechanical thromboprophylaxis alone. For the combined study arms (all retrospective and prospective study designs), the DVT rate for the additional pharmacological thromboprophylaxis arms was 0.71% (95% CI, 0.57%–0.89%) (43 trial arms, 13,869 participants, 63 events, *I* ^2^=0%). For mechanical thromboprophylaxis alone, the heterogeneity of 93% precluded quantitative synthesis and prompted further enquiry into between study differences.

A multivariate model was used to examine for significant moderators of outcome, and a Bonferroni correction applied to the significance threshold in light of the multiple analyses. In randomized trial arms of additional pharmacological thromboprophylaxis, moderators included in the model were symptomatic (imaging confirmed) versus asymptomatic (screen-detected) duplex scanning, single-dose versus extended pharmacological thromboprophylaxis, and compression therapy for 1 week or more. For mechanical thromboprophylaxis alone, pharmacological thromboprophylaxis was omitted from the model. None of the moderators in the model demonstrated any effect on DVT rate in the pharmacological thromboprophylaxis arms (*P*=0.97), nor mechanical thromboprophylaxis alone arms (*P*=0.13). Post hoc analysis revealed no difference in DVT rate between single-dose and extended pharmacological thromboprophylaxis (*P*=0.38), with a rate of 0.43% (95% CI, 0.22%–0.86%) for single-dose versus 0.75% (95% CI, 0.47%–1.19%) for extended pharmacological thromboprophylaxis (2 d or more).

### Pulmonary Emboli

There were 138 trial arms that reported PE as an outcome. The rate of PE in randomized study designs was 0.45% (95% CI, 0.09–2.35) (5 studies, 460 participants, 1 event, *I* ^2^=0%) for additional pharmacological thromboprophylaxis arms versus 0.23% (95% CI, 0.1%–0.52%) (28 studies, 4834 participants, 3 events, *I* ^2^=0%) for mechanical thromboprophylaxis alone. For the combined study arms, the rate of PE for the pharmacological thromboprophylaxis study arms was 0.14% (95% CI, 0.07%–0.28%) (26 studies, 9798 participants, 6 events, *I* ^2^=0%). For mechanical thromboprophylaxis alone in combined study arms, the rate of PE was 0.16% (95% CI, 0.15%–0.18%) (112 studies, 358,316 participants, 458 events, *I* ^2^=0%). The multivariate model demonstrated no significant moderators of outcome among study design and duration of compression.

### Endovenous Heat-induced Thrombosis

Of the studies reporting DVT rate, 16 documented the individual grade of endovenous heat-induced thrombosis (EHIT). As EHIT grade I and II are generally considered a spectrum of treatment effect and do not require treatment,^12^ only the rate of EHIT III and IV are reported. In the additional pharmacological thromboprophylaxis arms, the rate of EHIT grade III to IV was 0.35% (95% CI, 0.09–1.40) (3 studies, 822 participants, 5 events) versus 0.88% (95% CI, 0.28%–2.70%) (11 studies, 48,177 participants, 155 events) in the mechanical alone arms.

### Safety Endpoints

Major and minor bleeding were defined as per the International Society on Thrombosis and Haemostasis guidelines.^16^ Of the 12 trial arms that reported on bleeding after pharmacological thromboprophylaxis, there was 1 instance of major bleeding, associated with the coadministration of LMWH and ibuprofen.^17^ Other trials that considered minor bleeding rates noted a range from 0%^17–21^ up to 10%.^22–28^ There was no significant difference in bleeding events between 3 days of Fondaparinux versus Rivaroxaban, nor between Rivaroxaban 10 mg OD for 5 days versus 10 days.^23,24^ Of the 11 trial arms that included VTE-related mortality as an outcome, there was 1 VTE-related death that occurred in the mechanical thromboprophylaxis alone group after a PE from an iliofemoral DVT.^29^

### Risk of Bias

Studies were assessed for risk of bias using the Risk of Bias in Non-Randomised Studies of Interventions tool for nonrandomized studies of interventions and the Cochrane Risk of Bias tool for randomized trials (Table, Supplemental Digital Content 4, http://links.lww.com/SLA/E263). Randomized trials were generally at high or unclear risk of bias, primarily because of the lack of registered protocols, high rates of attrition, and potential for performance biases due to lack of blinding. For nonrandomized studies, there was a critical risk of selection bias through confounding by indication, as well as significant attrition bias and high potential for reporting bias.

### Quality Assessment

In the randomized trial arms, the quality of evidence for the rates of DVT and PE were judged as moderate in light of the risk of bias. The quality of evidence among nonrandomized studies was very low in light of the very serious risk of bias introduced by observational studies, confounding, and the degree of imprecision in the results.

## DISCUSSION

The true rate of VTE after endovenous varicose vein intervention has been an area of significant debate, ranging from 0% to 5.6% even in randomized trial arms.^30,31^ A previous review from 2018 reported a DVT event rate of 1.7%, including DVT and EHIT II to IV.^32^ Although data on pharmacological thromboprophylaxis regimes were collected in the review, the authors did not analyze the impact of anticoagulants on the rate of thrombotic complications. Furthermore, the authors analyzed only prospective data and adopted a random-effects model in light of statistically significant heterogeneity. Altogether, these reasons may explain the differences in the total observed rates of thrombotic complications, although it is reassuring that both the reviews report overall similar VTE rates.

Retrospective trial arms were included in this review, with the aim of generating the most comprehensive overview to date of thrombotic complications after endovenous varicose vein interventions. However, high heterogeneity introduced by the inclusion of retrospective trial arms, as indicated by an *I* ^2^ value of >90%, precluded quantitative synthesis by fixed or random effects. The heterogeneity has highlighted significant differences between prospective and retrospective studies, particularly the report from a large retrospective registry with over 250,000 participants.^2^ This registry reports a DVT rate of 3.1%^2^ and purports the reason for the higher than average rates to be registry data more closely reflecting real-world practice outside of a controlled clinical trial setting. However, the study fails to distinguish between EHIT and DVT during ultrasound follow-up, which severely limits its external validity; this, at least in part, explains the above average DVT rate. Even with exclusion of this trial, the *I* ^2^ value remains >80% for pooled prospective and retrospective studies, suggesting unaccounted confounding variables that preclude any further data pooling.

The latest European Society of Vascular Surgery guidelines suggest that perioperative anticoagulants should be considered for patients undergoing varicose vein surgery and a personalized risk assessment should be undertaken for individual patients.^12^ In the absence of prescriptive guidance, consensus studies have sought to ascertain how surgeons manage the risk of VTE after endovenous varicose vein intervention. The most recent consensus showed the greatest perceived risk factors for DVT after endovenous ablation were inherited thrombophilia, personal history of VTE, reduced mobility, obesity, previous malignancy and major surgery in last 12 weeks.^13^ In the United Kingdom, the Department of Health risk assessment tool is used to individually stratify the risk of VTE in every patient preoperatively. However, this scoring system fails to account for some of the strongest perceived risk factors identified by consensus. This study also demonstrated that the most commonly prescribed perioperative thromboprophylaxis regime was a single dose of LMWH for moderate-risk patients and 5 to 7 day course of LMWH for those at high risk. An alternative risk score is the Caprini score, which is widely validated for use in surgical patients.^33,34^ Risk stratification to determine thromboprophylaxis regime has been reported infrequently in the literature. Obi et al^35^ prescribed patients with a Caprini score >8 extended LMWH thromboprophylaxis for 7 days, with those at moderate risk (5 or more) receiving a single dose of unfractionated heparin perioperatively and low-risk patients receiving no pharmacological thromboprophylaxis. There were no differences in DVT rates between the risk groups, though the study was not powered to detect the small absolute risk reduction. Similarly, Knipp et al^36^ utilized the Caprini score in implementing a risk-adjusted protocol for no thromboprophylaxis, single-dose of unfractionated heparin or LMWH, or extended LMWH prophylaxis for 7 days. They demonstrated no difference in DVT rate before versus after the introduction of this risk stratification protocol. As the number of participants who received pharmacological thromboprophylaxis and DVT was not reported, the risk stratified arm could not be included in the quantitative synthesis. Altogether, the literature and guidelines reflect great uncertainty and inconsistency around risk stratification and appropriate pharmacological thromboprophylaxis, likely because of an absence of level I evidence. Randomized controlled trials are urgently needed.

The inherent risk of pharmacological thromboprophylaxis is the risk of bleeding. There was insufficient reporting of this outcome to perform a quantitative analysis, so a narrative review was performed. Qualitative studies have demonstrated reticence of patients to engage with thromboprophylaxis regimes, chiefly because of the lack of education about the risks of VTE, lack of involvement in the risk assessment process, and low acceptability of self-injection.^37^ Direct oral anticoagulant have been used for pharmacological thromboprophylaxis after surgery without increasing the major bleeding risk; however, minor bleeding events were more common.^38^ The data collected in this study support a similar hypothesis, with low rates of minor bleeding and just one instance of major bleeding across all the studies, due to NSAID and LMWH coadministration. Given the relative lack of safety data, it is imperative that primary safety endpoints such as bleeding and VTE-related mortality are reported by all future observational and interventional studies as per established international guidance.^16^

In this study, all endovenous treatment modalities were considered together, to enhance the amount of literature that could be analyzed. No significant difference in DVT rates for different endovenous closure modalities has been reliably demonstrated and post hoc analysis demonstrated no correlation between type of intervention and rate of DVT. In the same way, treatment of incompetent SSV and anterior accessory saphenous vein were included in quantitative analyses. Although some have discussed a trend towards a higher DVT rate when treating the SSV,^39^ the majority of trials have not documented such a phenomenon. Further post hoc analysis did not demonstrate any correlation between the treatment of great saphenous vein versus SSV and DVT rate.

### Limitations

The most significant limitation of this meta-analysis is the lack of direct head-to-head randomized trials comparing additional pharmacological thromboprophylaxis to mechanical measures alone. Therefore, network and standard meta-analysis were precluded, and a metaproportions approach was taken to aggregate the data from randomized trial arms and observational studies. The observational study arms introduced a significant amount of bias into the analysis, chiefly through confounding by indication. Trial arms in which pharmacological thromboprophylaxis was administered may have done so for a perceived higher risk of thrombosis in the cohort. Likewise, participants in the mechanical thromboprophylaxis alone trial arms are more likely to have been recruited to the trial because of their low perceived VTE risk. However, this bias is likely to diminish the effect size reported rather than increasing it. That is, the VTE rate may be overestimated for pharmacological in addition to mechanical thromboprophylaxis. This selection bias makes the quality of evidence for the pooled rate of DVT inherently weaker and explains the downgrading of evidence from high to moderate quality in the GRADE assessment. Although no direct head-to-head comparisons, the data extracted from randomized trial arms limits the level of confounding by indication and provides a less biased review of the pooled VTE rate for pharmacological in addition to mechanical thromboprophylaxis versus mechanical thromboprophylaxis alone. Thus, the randomized data are the most important findings presented.

Data were treated pragmatically; those studies that did not state the class duration or use of compression were still included in the analysis with the assumption that national and manufacturer guidance were followed. Overall, the reporting of compression therapy, including the class, duration, and adherence, was poor and, given all 3 of these variables were rarely reported in any study, the trial arms were still included without the description of mechanical thromboprophylaxis regime. Similarly, in the anticoagulation study arms, the differences in agent, dose, and duration may explain why no difference was observed in DVT rate between single-dose and extended thromboprophylaxis. Indeed, the heterogeneity in mechanical thromboprophylaxis reporting may account for a degree of the total observed heterogeneity. Compression data missingness in the retrospective cohort studies was 35% compared with 8% and 6% for prospective studies and randomized trial arms, respectively, and may give further reason for the very high heterogeneity observed in the combined prospective and retrospective studies for mechanical thromboprophylaxis alone that precluded meta-analysis.

## CONCLUSIONS

The rate of VTE after endovenous varicose vein intervention may be higher than previously anticipated and carries a small but significant morbidity and mortality. There is evidence that additional pharmacological thromboprophylaxis reduces the rate of DVT and it is recommended that anticoagulant agents are considered for endovenous varicose vein procedures. There is a strong likelihood of selection bias inherent within the analyzed studies; the pooled data from randomized trial arms are the most robust but are still limited by lack of head-to-head comparisons. Critical safety endpoints, such as major and minor bleeding, are generally underreported and raise the question of the most appropriate pharmacological thromboprophylaxis agent in the era of oral substitutes. Studies demonstrate highly variable VTE risk between participants and risk stratification using a validated scoring system may be useful for future randomized controlled trials to ensure equipoise to randomization and establish the efficacy and safety of this intervention.

## Supplementary Material
