# Supplementary material for: Systematic Review and Meta-analysis of the Additional Benefit of Pharmacological Thromboprophylaxis for Endovenous Varicose Vein Interventions
Source: Ann Surg. 2022 Oct 7;278(2):166–71. doi: 10.1097/SLA.0000000000005709 (PMC10321513; doi:10.1097/SLA.0000000000005709)
Supplement: Supplementary file 1 [file sla-278-0166-s001.docx]

**Supplemental Digital Content 1 – Search terms used to screen the literature.**

((varic* adj3 vein*) or varicosit* or varices).mp

AND

(((vein or venous or deep hospital acquired or hospital-acquired) adj3 thromb*) or (thromboembolic adj3 disease) or Pulmonary embolism or (PE or VTE or DVT)).mp

AND

((Vascular or endovascular or foam or venous or endovenous or vein or varicos* or saphenofemoral or thermal or endothermal saphenopopliteal or saphenous or junction or radiofrequency or mechanical or chemical or mechanochemical or cyanoacrylate) adj3 (ablation or la?er or sclerotherapy or surgery or disconnect* or glue or treatment or procedure or intervention)).mp
