# Supplementary material for: Systematic Review and Meta-analysis of the Additional Benefit of Pharmacological Thromboprophylaxis for Endovenous Varicose Vein Interventions
Source: Ann Surg. 2022 Oct 7;278(2):166–71. doi: 10.1097/SLA.0000000000005709 (PMC10321513; doi:10.1097/SLA.0000000000005709)
Supplement: Supplementary file 2 [file sla-278-0166-s002.docx]

**Supplemental Digital Content 2 - PRISMA flow diagram of included studies.**

**Identification of studies via databases and registers**

Records removed *before screening*:

Duplicate records removed (n = 513)

Records identified from:

Medline+Embase (n = 2164)

Cochrane Library (n=30)

Registers (n = 0)

**Identification**

Records screened

(n = 1681)

Records excluded during title and abstract screening

(n = 1324)

Reports sought for retrieval

(n = 357)

Reports not retrieved

(n = 11)

**Screening**

Reports excluded (n=125):

Full text not available in English (n = 23) yellow

Wrong study design (n = 11) blue

Wrong population (n = 8) purple

Wrong intervention (n = 23) orange

Wrong outcomes (n = 30) green

Data previously published elsewhere (n = 8) red

Insufficient participants (n = 5)

Inability to extract separate data for intervention arm (n = 6) white

Reports assessed for eligibility

(n = 346)

Studies included in review

(n = 221)

Reports of included studies

(n = 221)

**Included**
