# Supplementary material for: Systematic Review and Meta-analysis of the Additional Benefit of Pharmacological Thromboprophylaxis for Endovenous Varicose Vein Interventions
Source: Ann Surg. 2022 Oct 7;278(2):166–71. doi: 10.1097/SLA.0000000000005709 (PMC10321513; doi:10.1097/SLA.0000000000005709)
Supplement: Supplementary file 3 [file sla-278-0166-s003.docx]

**Supplemental Digital Content 3– The summary data collected from each study included in the meta-analysis.**

| **Author** | **Year** | **Truncal Vein** | **Study Design** | **Intervention** | **CEAP** | **Number of participants** | **EHIT any grade** | **DVT** | **PE** | **Compression Description** | **Antithrombotic regime** | **Antithrombotic details** | **Symptomatic scanning** | **Timing of scanning** |
| --- | --- | --- | --- | --- | --- | --- | --- | --- | --- | --- | --- | --- | --- | --- |
| Abbassi-Ghadi et al.^S1^ | 2013 | GSV | Prospective cohort study | UGFS | 2 - 6 | 213 | Not stated | 1 (0.5%) | 1 (0.5%) | Not stated | Nil | Nil | Symptomatic | 6 - 8 weeks |
| Agus et al.^S2^ | 2006 | GSV, SSV | Prospective cohort study | EVLA | 2 - 6 | 1050 | Not stated | 0 (0.0%) | 0 (0.0%) | Not stated | Nil | Nil | Symptomatic | 36 months |
| Almeida et al.^S3^ | 2009 | GSV | RCT | EVLA, RFA | Most C2 | 69 | Not stated | 1 (1.4%) | 0 (0.0%) | Compression bandages and class II compression stockings for 24-72 hours, then stockings for 2 weeks | Not stated | Not stated | Asymptomatic | 48 hour and 1 month |
| Arslan et al.^S4^ | 2017 | GSV | Prospective cohort study | EVLA | Not stated | 400 | Not stated | 4 (1.0%) | 0 (0.0%) | Compressive stockings 30-40mmHg were worn for 3 months | Not stated | Not stated | Asymptomatic | day 1, week 1, and 1, 6, 12, 24, 36 and 48 months |
| Atlin et al.^S5^ | 2015 | GSV | Retrospective cohort study | EVLA | 1 - 5 | 200 | Not stated | 0 (0.0%) | 0 (0.0%) | Elastic compression for 24 hours then above knee compression for 4 weeks. | Not stated | Not stated | Asymptomatic | 1 week, 1 month, 6 months |
| Aurshina et al.^S6^ | 2018 | GSV, SSV, ASV | Retrospective cohort study | RFA | 2 - 6 | 362 | 8 (2.2%) | 1 (0.3%) | Not stated | Not stated | Not stated | Not stated | Symptomatic | 2 days |
| Baccellieri et al.^S7^ | 2021 | SSV | Prospective cohort study | MOCA | 2 - 6 | 60 | Not stated | 0 (0.0%) | 0 (0.0%) | 23mmHg compression stocking post-op then to be worn during the day for 2 weeks | Not stated | Not stated | Asymptomatic | 1, 6 and 12 months |
| Bademci et al.^S8^ | 2018 | GSV | Retrospective cohort study | Cyanoacrylate glue | 2 - 4 | 50 | Not stated | 0 (0.0%) | 0 (0.0%) | Not stated | Not stated | Not stated | Asymptomatic | 1, 6 and 12 months |
| Barker et al.^S9^ | 2017 | Unspecified | Retrospective cohort study | EVLA, RFA, UGFS | Not stated | 81,970 | Not stated | 420 (0.5%) | Not stated | Not stated | Not stated | Not stated | Symptomatic | Analysis of hospital episodes statistics data |
| Belcaro et al.^S10^ | 2010 | GSV | RCT | UGFS | 2 | 32 | Not stated | 0 (0.0%) | Not stated | Graduated compression stockings were applied over the elastic bandages. | Not stated | Not stated | Symptomatic | 1, 5 and 10 years |
| Benarroch-Gampel et al.^S11^ | 2013 | GSV, SSV | Retrospective cohort study | EVLA, RFA | 2 - 6 | 3874 | Not stated | 61 (1.6%) | 2 (0.0%) | 30-40mmHg compression stockings for 48-72 hours | Single shot | 222 with C6 disease, given single shot of LMWH and compression lifelong/until ulcer healed | Symptomatic | Symptomatic: timing of duplex unknown from database and hospital episode data analysed |
| Bergan et al.^S12^ | 2006 | Truncal but not specified | Prospective cohort study | UGFS | 2- 6 | 261 | Not stated | 6 (2.3%) | 0 (0.0%) | Compression bandage for 48 hours then class III compression stocking in the daytime for 14 days | Not stated | Not stated | Asymptomatic | 1, 7, 14 and 30 days |
| Beteli et al.^S13^ | 2018 | GSV | RCT | RFA | 2 - 5 | 42 | 1 (2.4%) | 1 (2.4%) | 0 (0.0%) | Compression bandages for 48 hours then elastic stockings during the follow up period | Not stated | Not stated | Asymptomatic | 1 week, 3 and 6 months |
| Biemans et al.^S14^ | 2013 | GSV | RCT | EVLA, UGFS | 2 - 5 | 155 | Not stated | 0 (0.0%) | 0 (0.0%) | Compression bandage for 48 hours then stockings for 2 weeks | Not stated | Not stated | Symptomatic | 3 and 12 months |
| Bisang et al.^S15^ | 2012 | GSV, SSV | Retrospective cohort study | RFA | 2 - 6 | 73 | Not stated | 0 (0.0%) | 1 (1.4%) | 20-30mmHg compression stockings for 24 hours | Nil | Nil | Asymptomatic | Post-procedural then 12.2 months (1 - 19 range) |
| Bishawi et al.^S16^ | 2014 | GSV | Prospective cohort study | MOCA | 2 - 6 | 126 | Not stated | 0 (0.0%) | 0 (0.0%) | 98% patient sent home with compression stockings | Nil | Nil, 28 patients taking warfarin/ clopidogrel | Asymptomatic | 1 week, 3 months, 6 months |
| Bitargil et al.^S17^ | 2020 | GSV | Retrospective cohort study | RFA | 1 - 4 | 128 | Not stated | 1 (0.8%) | Not stated | Not stated | Not stated | Not stated | Symptomatic | Closure rate assessed at 3 months only |
| Blaise et al.^S18^ | 2010 | GSV | RCT | UGFS | 2 - 5 | 143 | Not stated | 3 (2.1%) | 0 (0.0%) | Compression of 34–49 mmHg was achieved by applying an adhesive foam bandage for 3 days, then class 2 elastic compression stocking for 15 days during the day. | Not stated | Not stated | Asymptomatic | 8 days, 6 weeks, 3 and 6 months, 1, 2 and 3 years |
| Boersma et al.^S19^ | 2013 | SSV | Prospective cohort study | MOCA | 2 - 6 | 50 | Not stated | 0 (0.0%) | Not stated | Compression stockings 30–40 mmHg continuously for the first 24 h and during the daytime for the next 2 weeks | Nil | Nil, patients on anticoagulation excluded | Symptomatic | 6 weeks and 1 year |
| Bootun et al.^S20^ | 2014 | GSV | RCT | MOCA, RFA | Not stated | 119 | Not stated | 1 (0.8%) | Not stated | Stockings were worn for two weeks post-procedure | Not stated | Not stated | Asymptomatic | 1 month |
| Borghese et al.^S21^ | 2021 | GSV | Prospective cohort study | RFA | 1 - 5 | 135 | Not stated | 0 (0.0%) | 0 (0.0%) | Elastic compression stockings 30-40mmhg 24 hours then 20-30mmHg 1 week | Extended | Subcutaneous dose of prophylactic LMWH for 8 days post-procedure | Asymptomatic | 1 month |
| Boros et al.^S22^ | 2008 | GSV | Retrospective cohort study | RFA | Not stated | 142 | Not stated | 5 (3.5%) | Not stated | Not stated | Nil | Nil, 15 were taking anticoagulants at the time of procedure | Asymptomatic | 24 hours then 1 month |
| Bozkurt et al. ^S23^ | 2016 | GSV | Prospective cohort study | Cyanoacrylate glue, EVLA | 2 - 4 | 310 | Not stated | 0 (0.0%) | 0 (0.0%) | In CAA group a single adhesive bandage is applied to the sheath introduction site; neither compression stockings nor compression bandages are used. Varicose stockings were prescribed for 10 days after EVLA procedure | Not stated | Not stated | Asymptomatic | Day 3, 1, 6, and 12 months |
| Bozoglan et al.^S24^ | 2016 | GSV | RCT | EVLA, RFA | 3 - 4 | 60 | Not stated | 0 (0.0%) | 0 (0.0%) | An elastic bandage was applied for 2 days to the leg receiving the procedure. Compression socks were subsequently recommended for 3 months | Not stated | Not stated | Asymptomatic | Week 1, 1, 3 and 6 months |
| Bradbury et al.^S25^ | 2010 | GSV, SSV, ASV | Prospective cohort study | UGFS | 2 - 6 | 977 | Not stated | 3 (0.3%) | 1 (0.1%) | Full length class II stocking for 5-7 days then worn 24 hours a day for 7 days, then in the daytime for 2 weeks | Single shot | Single shot of 20-40mg enoxaparin; 12 took warfarin and no anticoagulation/antiplatelets were stopped for the procedure | Asymptomatic | 1 and 12 months |
| Brown et al.^S26^ | 2021 | GSV, SSV | Retrospective cohort study | EVLA | 2 | 3375 | Not stated | 27 (0.8%) | Not stated | Not stated | Not stated | Not stated | Unclear | Not stated |
| Cabrera et al.^S27^ | 2004 | GSV | Retrospective cohort study | UGFS | 6 | 116 | Not stated | 0 (0.0%) | 0 (0.0%) | The treatment is complemented with compression bandaging 23mmHg stocking for 7-15 days | Not stated | Not stated | Symptomatic | 6 months; but re-foamed at 2-4 weeks |
| Cabrero Fernandez et al.^S28^ | 2017 | GSV | Prospective cohort study | RFA | 2 - 5 | 257 | Not stated | 1 (0.4%) | 0 (0.0%) | Elastic support with a medium-compression long stocking 22-29 mmHg | Single/ Extended | All received a prophylactic dose of subcutaneous LMWH (usually 40 mg of enoxaparin or 3500 units of bemiparin daily) during the 7 days after surgery | Asymptomatic | 1, 6 and 12 months |
| Calcagno et al.^S29^ | 2009 | GSV, SSV | Prospective cohort study | RFA | Not stated | 338 | Not stated | 0 (0.0%) | Not stated | Not stated | Not stated | Not stated | Asymptomatic | Day 2-5 and 6 months |
| Campos Gomes et al. ^S30^ | 2020 | GSV | RCT | UGFS | 2 - 6 | 69 | Not stated | 2 (2.9%) | 0 (0.0%) | 72 limbs had elastic compression, 63 did not | Nil | Nil | Asymptomatic | 3 weeks then 3 months |
| Can Caliskan et al.^S31^ | 2013 | GSV, SSV | Prospective cohort study | EVLA | Not stated | 30 | Not stated | 0 (0.0%) | 0 (0.0%) | Compression stockings for one week then during the day only for 3 weeks | Not stated | Not stated | Symptomatic | 6 weeks |
| Carradice et al.^S32^ | 2011 | GSV | RCT | EVLA | 2 - 6 | 137 | Not stated | 0 (0.0%) | 0 (0.0%) | Gauze and elastic compression dressings applied. These were later replaced by a thigh-length antiembolism stocking, which were advised to wear for a total of 6 weeks. | Not stated | Not stated | Asymptomatic | 1 week, 6 weeks, 3 months, 1 year, 2 years |
| Carruthers et al.^S33^ | 2014 | GSV, SSV | Retrospective cohort study | EVLA, RFA | Not stated | 1786 | Not stated | 28 (1.6%) | Not stated | Not stated | Not stated | Not stated | Unclear | Unclear, data were collected from a national registry and scanning frequency is not reported. |
| Casana et al.^S34^ | 2018 | GSV, SSV | Retrospective cohort study | RFA | 2 - 5 | 1080 | Not stated | 3 (0.3%) | 0 (0.0%) | Not stated | Not stated | Not stated | Asymptomatic | 1 week, 1, 6, 12, 24, 36 months |
| Cavezzi et al.^S35^ | 2002 | GSV, SSV, ASV | Prospective cohort study | UGFS | 2 - 6 | 177 | 2 (1.1%) | 0 (0.0%) | Not stated | Compression pads then 20-30 or 30-40mmHg stocking applied. | Not stated | Not stated | Asymptomatic | 1 month |
| Cavezzi et al.^S36^ | 2017 | GSV | Prospective cohort study | UGFS | Not stated | 82 | Not stated | 0 (0.0%) | 0 (0.0%) | 35mmHg compression stockings for 7 days then class 1 compression stocking for 30 days in daytime | Single shot | Single injection of prophylactic LMWH | Asymptomatic | 40 days, 6, 12, 36 months |
| Chaar et al ^S37^ | 2011 | GSV, SSV, ASV | Retrospective cohort study | EVLA | Not stated | 885 | 8 (0.9%) | 2 (0.2%) | 0 (0.0%) | Long stretch compression bandage for 24 hours then 20-30mmHg stocking during the day for 1 week | Nil | Nil | Asymptomatic | 2 weeks |
| Chan et al.^S38^ | 2020 | GSV | Prospective cohort study | Cyanoacrylate glue | 6 | 37 | Not stated | 1 (2.7%) | 0 (0.0%) | All received post-op compression until healed | Not stated | Not stated | Asymptomatic | 1 week, 3 months, 6 months, 12 months |
| Chan et al.^S39^ | 2017 | GSV | Retrospective cohort study | MOCA | 3 - 6 | 55 | 2 (3.6%) | 0 (0.0%) | Not stated | All were advised to wear full-length compression stockings for at least 1 month. | Not stated | Not stated | Asymptomatic | 1 week, 1, 6 and 12 months |
| Chandler et al.^S40^ | 2000 | GSV | Prospective cohort study | EVLA | 0 - 6 | 223 | 3 (1.3%) | 1 (0.4%) | 1 (0.4%) | Short stretch elastic bandage then knee-length compression stockings for 3 weeks | Not stated | Not stated | Asymptomatic | 1 and 6 weeks, 6 months, 1 year |
| Chang et al. ^S41^ | 2021 | Truncal but not specified | Retrospective cohort study | RFA | Not stated | 1323 | Not stated | 24 (1.8%) | 2 (0.2%) | Not stated | Not stated | Not stated | Symptomatic | Patient data was identified and followed up retrospectively til death |
| Chen et al. ^S42^ | 2013 | GSV | Prospective cohort study | EVLA | 2 - 4 | 31 | Not stated | 0 (0.0%) | 0 (0.0%) | Compression bandaging for 48 hours then class II graduated compression stockings | Not stated | Not stated | Asymptomatic | 1, 12, 18 months |
| Chi et al. ^S43^ | 2014 | GSV, SSV | Prospective cohort study | EVLA | Not stated | 360 | 18 (5.0%) | 1 (0.3%) | 0 (0.0%) | 20-30mmHg compression hosiery for 7 days then during waking hours for another week | Not stated | Not stated | Asymptomatic | 1 week |
| Cho et al. ^S44^ | 2020 | Truncal but not specified | Prospective cohort study | Cyanoacrylate glue | Not stated | 191 | 11 (5.8%) | 0 (0.0%) | 0 (0.0%) | Not stated | Not stated | Not stated | Asymptomatic | 1 week, 3 months, 6 months, 12 months |
| Choi et al. ^S45^ | 2013 | GSV, SSV | Retrospective cohort study | RFA | 2 - 6 | 148 | 2 (1.4%) | 1 (0.7%) | Not stated | Elastic bandage for 24 hours then compression stocking for 2 weeks | Not stated | Not stated | Asymptomatic | 1 week and 6 months |
| Christenson et al. ^S46^ | 2010 | GSV | RCT | EVLA | 2 - 6 | 100 | Not stated | 0 (0.0%) | Not stated | Compression bandaging for 48 hours then class II compression stocking for 3 weeks | Extended | Enoxaparin 20-40mg for 10 days (depending on weight) | Asymptomatic | 12 days, 1 and 2 years |
| Ciostek et al.^S47^ | 2015 | GSV, SSV | Prospective cohort study | MOCA | 2 - 6 | 39 | Not stated | 0 (0.0%) | 0 (0.0%) | Elastic bandage or class 2 compression stocking applied | Not stated | Not stated | Asymptomatic | 1, 3, 6 and 12 months |
| Creton et al.^S48^ | 2010 | GSV | Prospective cohort study | RFA | 2 - 3 | 225 | 0 (0.0%) | 0 (0.0%) | 0 (0.0%) | Not stated | Not stated | Not stated | Asymptomatic | 3 days, 3 months, 6 months, and 1 year |
| Cuffolo et al.^S49^ | 2019 | GSV, SSV | Prospective cohort study | UGFS | 6 | 336 | Not stated | 8 (2.4%) | Not stated | Full length compression stockings for 6 days then worn for a further 2 weeks | Not stated | Not stated | Symptomatic | 6 weeks |
| Darvall et al.^S50^ | 2009 | SSV | Prospective cohort study | UGFS | 2 - 5 | 86 | Not stated | 1 (1.2%) | Not stated | Bandaging for 5–10 days, then a class II stocking for a further 3 weeks | Not stated | Not stated | Asymptomatic | 1, 6 and 12 months |
| De Aguiar et al.^S51^ | 2020 | GSV, SSV | Retrospective cohort study | UGFS | 2 - 6 | 2616 | Not stated | 7 (0.3%) | 8 (0.3%) | Not stated | Not stated | Not stated | Unclear | Only mentions those with <60 days follow up were excluded |
| De Araujo et al.^S52^ | 2016 | GSV | Prospective cohort study | EVLA | 2 - 5 | 36 | 1 (2.8%) | 0 (0.0%) | Not stated | Compressive bandages then 20-30mmHg stockings for 7 days | Not stated | Not stated | Asymptomatic | 3-5 days, 1, 6 and 12 months |
| De Oliveira et al.^S53^ | 2018 | GSV | Prospective cohort study | UGFS | 4 - 6 | 33 | 1 (3.0%) | 0 (0.0%) | 0 (0.0%) | Inelastic compression bandages for 3 days then 30-40mmHg compression stockings for 3 months | Not stated | Not stated | Asymptomatic | 7, 15, 30, 60, 90 days |
| Desmyttre et al. ^S54^ | 2007 | GSV | Prospective cohort study | EVLA | 2 | 511 | Not stated | 0 (0.0%) | 0 (0.0%) | Compression bandage for 24 hours then class III compression stocking during the day for 3 weeks | Not stated | Not stated | Asymptomatic | 1 day, 1 week, 1 and 6 months, 1, 2, 3 and 4 years |
| Desmyttre et al.  ^S55^ | 2010 | SSV | Prospective cohort study | EVLA | 2 - 4 | 128 | Not stated | 0 (0.0%) | 0 (0.0%) | Compression bandage for 24 hours then class III compression stocking for 3 weeks | Not stated | Not stated | Symptomatic | 12, 24, 36 months |
| Disselhoff et al.^S56^ | 2005 | GSV | Prospective cohort study | EVLA | 2 - 6 | 85 | Not stated | 0 (0.0%) | 0 (0.0%) | Graduated compression stocking 20-30mmHg for 1 week | Nil | Nil | Symptomatic | 6 weeks, then 3, 6, 12 and 24 months |
| Disselhoff et al.^S57^ | 2008 | GSV | RCT | EVLA | 2 | 60 | Not stated | 0 (0.0%) | 0 (0.0%) | Graduated compression stocking 20-30mmHg for 1 week | Not stated | Not stated | Symptomatic | 6, 12 and 24 months |
| Doganci et al.^S58^ | 2010 | GSV | RCT | EVLA | 2 - 4 | 60 | Not stated | 0 (0.0%) | 0 (0.0%) | Compression bandage for 24 hours then graduated compression stockings for 7 days | Nil | Nil | Asymptomatic | 2 and 7 days, 1, 2, 3 and 6 months |
| Dunn et al.^S59^ | 2006 | GSV | Prospective cohort study | RFA | 1 - 6 | 68 | 0 (0.0%) | 0 (0.0%) | Not stated | Compression garments for 2 weeks | Not stated | Not stated | Asymptomatic | 3 days, 6 months |
| ElKaffas et al.^S60^ | 2011 | GSV | RCT | RFA | 2 - 5 | 90 | Not stated | 0 (0.0%) | 0 (0.0%) | Class II above knee compression stocking that was worn for 72 hours | Not stated | Not stated | Asymptomatic | 1 week, 1, 6, 12, 18 and 24 months |
| Ergenoglu et al.^S61^ | 2010 | GSV | Retrospective cohort study | EVLA | 2 - 5 | 43 | Not stated | 0 (0.0%) | 0 (0.0%) | Compression stockings for 6 weeks | Nil | Nil | Asymptomatic | 1 week, 3, 6, 12 months |
| Ergenoglu et al.^S62^ | 2011 | GSV | Prospective cohort study | EVLA | 2 - 5 | 98 | Not stated | 0 (0.0%) | 0 (0.0%) | Elastic compression bandage for 48 hours then class II compression stocking for 6 weeks during the day | Not stated | Not stated | Asymptomatic | 3, 6 and 12 months |
| Eroglu et al.^S63^ | 2018 | GSV, SSV | RCT | EVLA, RFA, Cyanoacrylate glue | 2 - 6 | 525 | Not stated | 1 (0.2%) | 0 (0.0%) | Elastic compression stocking for 2 days then class I compression for 1 month | Nil | Nil | Asymptomatic | 2 days, 1, 6, 12, 24 months |
| Fernandez et al.^S64^ | 2008 | GSV, SSV | Prospective cohort study | EVLA | 2 - 6 | 1559 | Not stated | 2 (0.1%) | 0 (0.0%) | Low stretch elastic bandage for 2 days then graduated support stockings at 15-20mmHg for 1 month in the day | Nil | Nil | Asymptomatic | 1 week, 1 month, 3 months, yearly |
| Fernando et al.^S65^ | 2014 | GSV | Prospective cohort study | EVLA | 2 - 6 | 319 | 2 | 0 (0.0%) | 0 (0.0%) | Below-knee thromboembolic deterrent stocking for an unspecified time period | Nil | Nil | Asymptomatic | 1 month |
| Fischer et al.^S66^ | 2021 | GSV | RCT | EVLA | 2 - 5 | 150 | Not stated | 0 (0.0%) | 0 (0.0%) | Group A no compression (n=48) therefore excluded., Group B graduated compression stocking (23–32 mmHg) for 7 days, group C graduated compression stocking (23–32 mmHg) for 28 days | Extended | Prophylactic anticoagulation was given for 7 days | Asymptomatic | 7 and 28 days |
| Flessenkamper et al.^S67^ | 2013 | GSV | RCT | EVLA | 1 - 4 | 142 | Not stated | 1 (0.7%) | Not stated | Not stated | Not stated | Not stated | Asymptomatic | Day 1 and 2, 6, 12, 24 months |
| Gabriel et al.^S68^ | 2012 | GSV, SSV | Retrospective cohort study | EVLA | 2 - 6 | 45 | 0 (0.0%) | 0 (0.0%) | 0 (0.0%) | Not stated | Extended | Long-term warfarin | Symptomatic | Scanned at 48 - 72 hours then hospital notes review |
| Gale et al.^S69^ | 2010 | GSV | RCT | EVLA, RFA | Not stated | 94 | 0 (0.0%) | 1 (1.1%) | Not stated | Compression bandage for 72 hours then 20-30mmHg stockings for 2 weeks | Not stated | Not stated | Asymptomatic | 1 week, 1 month and 1 year |
| Garcia-Madrid et al.^S70^ | 2013 | GSV | Prospective cohort study | RFA | Not stated | 67 | Not stated | 0 (0.0%) | Not stated | Elastic bandage for 24 hours then class II compression stockings for 1 week | Extended | Prophylactic LMWH for 6 days post-op | Asymptomatic | Day 4, 1, 3 and 6 months |
| Gibson et al.^S71^ | 2017 | GSV | RCT | UGFS | 2 - 5 | 73 | 3 (4.1%) | 7 (9.5%) | 0 (0.0%) | Short stretch bandage plus 30-40mmHg compression stockings for 48 hours then compression stockings for 12 days | Not stated | Not stated | Asymptomatic | Week 4 and 12 |
| Gibson et al.^S72^ | 2007 | SSV | Prospective cohort study | EVLA | 2 - 6 | 126 | 11 (8.7%) | 0 (0.0%) | Not stated | Class II compression stockings for 2 days then in daytime for 2 weeks | Nil | Nil | Asymptomatic | 2-4 days, 2-11 months |
| Gifford et al.^S73^ | 2014 | GSV | Retrospective cohort study | EVLA, RFA | 1 - 6 | 61 | 0 (0.0%) | 0 (0.0%) | Not stated | Compression garments but duration not specified | Not stated | Not stated | Asymptomatic | 1 day then 6 months |
| Gillet et al.^S74^ | 2016 | GSV, SSV, ASV | Prospective cohort study | UGFS | 1 - 6 | 418 | Not stated | 2 (0.5%) | Not stated | Elastic compression in 52-58% | Nil | Nil, anticoagulant therapy continued in 2.9-6.3% | Asymptomatic | 1 month |
| Gillet et al.^S75^ | 2009 | GSV, SSV | Prospective cohort study | UGFS | 2 - 6 | 1025 | Not stated | 10 (1.0%) | 1 (0.1%) | Elastic compression of 15mmHg applied in 72.3% | Not stated | Not stated | Asymptomatic | Day 8, Day 30 |
| Gillet et al.^S76^ | 2014 | SSV | Prospective cohort study | UGFS | 2 - 6 | 331 | 4 (1.2%) | 7 (2.1%) | 0 (0.0%) | Elastic compression of >15mmHg applied for 1 week at end of procedure in 84.6% | Nil | Nil | Asymptomatic | Day 8, Day 30 |
| Gohel et al. ^S77^ | 2018 | GSV, SSV | RCT | EVLA, RFA, MOCA, Cyanoacrylate glue | 6 | 450 | 3 (0.7%) | 9 (2.0%) | 0 (0.0%) | Multilayer elastic compression (two to four layers), short-stretch compression, and compression hosiery | Not stated | Not stated | Symptomatic | 6 weeks |
| Golbasi et al.^S78^ | 2015 | GSV, SSV | Prospective cohort study | EVLA | 1 - 6 | 552 | 2 (0.4%) | 0 (0.0%) | 0 (0.0%) | Elastic adhesive bandage for 2-4 days then class II full length graduated support stocking for 3-5 weeks in the day | Nil | Nil | Asymptomatic | 1, 6 months and 1, 2 years |
| Hamel-Desnos et al.^S79^ | 2009 | GSV, SSV | Retrospective cohort study | EVLA | 2 - 6 | 1422 | Not stated | 5 (0.4%) | 1 (0.0%) | Most wore 16-20mmHg compression stockings for 1 week - 1 month | Extended | Received a mean six-day LMWH for a median of five post-procedure days (range 3–11 | Asymptomatic | 1 and 6 months |
| Hamel-Desnos et al.^S80^ | 2009 | GSV, SSV | RCT | UGFS | 1 - 6 | 54 | Not stated | 0 (0.0%) | 0 (0.0%) | Compression stockings at 15-20mmHg for 4 weeks in the day | Single shot | Nadroparin 4000 IU single shot vs warfarin at 1mg/day for 10 days prior to treatment, at which point sclerotherapy was carried out | Asymptomatic | 4 weeks |
| Haqqani et al.^S81^ | 2011 | GSV | Prospective cohort study | RFA | 2 - 5 | 73 | 2 (2.7%) | 0 (0.0%) | Not stated | Compression dressing for 48 hours then compression shorts and stockings at 20-30mmHg | Single shot | Single shot subcutaneous heparin 3000-5000 units and post-op aspirin | Asymptomatic | 5-7 days and 1 month |
| Harlander-Locke et al.^S82^ | 2013 | SSV | Prospective cohort study | UGFS | 2 - 6 | 76 | 2 (2.6%) | 0 (0.0%) | 0 (0.0%) | Compressive bandage for 24-72 hours then compression recommended for 1-2 weeks | Nil | Nil | Asymptomatic | 48 - 72 hours |
| Hingorani et al.^S83^ | 2004 | GSV | Prospective cohort study | RFA | 2 - 6 | 66 | 11 (16.7%) | 1 (1.5%) | 0 (0.0%) | Elastic bandage for 48 hours | Nil | Nil, 7 took aspirin, 1 took warfarin, 1 took clopidogrel | Asymptomatic | 2 and 30 days |
| Hirokawa et al.^S84^ | 2015 | GSV, SSV | RCT | EVLA | 2 - 4 | 113 | 5 (4.4%) | 2 (1.8%) | 0 (0.0%) | Compression bandage for 24 hours then compression stocking for 5 days, then below knee compression stockings for 4 weeks | Not stated | Not stated | Asymptomatic | 1 and 5 days, 2, 4 and 12 weeks |
| Holewijn et al.^S85^ | 2018 | GSV | RCT | MOCA, RFA | 2 - 5 | 209 | Not stated | 1 (0.5%) | 0 (0.0%) | 20-30mmHg compression stocking for 24 hours then daily for 2 weeks | Nil | Nil | Asymptomatic | 30 days, 1, 2 years |
| Huisman et al.^S86^ | 2009 | SSV | Prospective cohort study | EVLA | 1 - 6 | 150 | Not stated | 0 (0.0%) | Not stated | Compression stockings for 72 hrs | Not stated | Not stated | Symptomatic | 3 months |
| Itoga et al.^S87^ | 2019 | Truncal but not specified | Retrospective cohort study | EVLA, RFA |  | 256,999 | Not stated | 8,203 (3.2%) | 423 (0.2%) | Not stated | Not stated | Not stated | Asymptomatic | Follow up scan within 30 days |
| Izumi et al.^S88^ | 2016 | GSV, SSV | Retrospective cohort study | EVLA | 4 - 6 | 119 | Not stated | 0 (0.0%) | 0 (0.0%) | Elastic compression for 24 hours then compression at 30-40mmHg for 4 weeks. | Not stated | Not stated | Asymptomatic | 1 week, 1, 3, 6, 12, 18, 24, 30 months |
| Jacobs et al.^S89^ | 2014 | GSV, SSV | Prospective cohort study | RFA | Not stated | 277 | 11 (4.0%) | 2 (0.7%) | Not stated | Gauze wraps and an elastic bandage from the toes to the groin. | Nil | Nil, 12 took warfarin and 47 took aspirin | Asymptomatic | 2 weeks |
| Janee D'Orthee et al.^S90^ | 2010 | SSV | Retrospective cohort study | EVLA | Not stated | 63 | Not stated | 0 (0.0%) | 0 (0.0%) | 20-30mmHg or 30-40mmHg stockings for 1 week | Not stated | Not stated | Asymptomatic | 1-2 weeks, 3-6 months, 12 months |
| Jimenez et al.^S91^ | 2021 | GSV, SSV | Prospective cohort study | UGFS | 2 - 6 | 49 | 1 (2.0%) | 1 (2.0%) | 0 (0.0%) | Compression stockings at 20-30mmHg for 14 days | Nil | Nil | Asymptomatic | 48-72 hours |
| Jiminez et al.^S92^ | 2021 | GSV, SSV | Prospective cohort study | UGFS | 3 - 6 | 122 | 2 (1.6%) | 2 (1.6%) | 0 (0.0%) | 20-30mmHg stockings up to thigh for 14 days | Nil | Nil, 18 took chronic anticoagulation | Asymptomatic | 48-72 hours |
| Jung et al.^S93^ | 2008 | GSV, SSV | Retrospective cohort study | EVLA | 2 - 6 | 148 | Not stated | 0 (0.0%) | 0 (0.0%) | Eccentric compression bandage for 24-48 hours after procedure then full length class II compression stocking for 4-6 weeks | Not stated | Not stated | Asymptomatic | Week 1 and 12 |
| Kane et al.^S94^ | 2014 | GSV, SSV | Retrospective cohort study | EVLA | 2 - 6 | 528 | 24 (4.5%) | 0 (0.0%) | 0 (0.0%) | Compression bandage | Not stated | Not stated | Asymptomatic | 1 week |
| Kapoor et al.^S95^ | 2010 | GSV | Retrospective cohort study | RFA | 1 - 4 | 100 | 0 (0.0%) | 0 (0.0%) | Not stated | Compression bandage applied along with a compression stocking for a period of 2 weeks | Not stated | Not stated | Asymptomatic | 2 weeks, 3 months, 1 year |
| Karathanos et al.^S96^ | 2021 | GSV | Prospective cohort study | EVLA, RFA | 2 - 6 | 162 | 5 (3.1%) | 0 (0.0%) | Not stated | Compression bandage for 48 hours then elastic compression stocking for 1 week | Not stated | Not stated | Asymptomatic | 7 and 30 days, 1 year |
| Keo et al.^S97^ | 2019 | GSV, SSV, ASV | Retrospective cohort study | EVLA | 2 - 6 | 829 | 38 (4.6%) | 3 (0.4%) | 0 (0.0%) | Compressive bandage 24 - 72 hours then class 2 compression in the day for up to 1 week | Extended | Rivaroxaban 10mg OD 3 or Fondaparinux 2.5mg OD 3-10 days post-op | Asymptomatic | 4 weeks |
| Keo et al.^S98^ | 2017 | GSV, SSV | Retrospective cohort study | EVLA | 2 - 6 | 391 | 15 (3.8%) | 3 (0.8%) | 0 (0.0%) | 20-30mmHg GCS for 3 day, then class 2 compression during the day for a further 4 days | Extended | Rivaroxaban 10mg OD 3 days or Fondaparinux 2.5mg OD 3 days | Asymptomatic | 1 and 4 weeks |
| Keo et al.^S99^ | 2019 | GSV, SSV, ASV | Retrospective cohort study | EVLA | 2 - 6 | 793 | 43 (5.4%) | 3 (0.4%) | 0 (0.0%) | Compressive bandage 24 - 72 hours then class 2 compression for up to 1 week | Extended | Rivaroxaban 10mg OD 3 days or Fondaparinux 2.5mg OD 3 days or Rivaroxaban 10mg OD 10 days | Asymptomatic | 1 and 4 weeks |
| Khan Karl et al.^S100^ | 2019 | GSV, SSV | Prospective cohort study | UGFS | 2 - 6 | 662 | Not stated | 0 (0.0%) | Not stated | Multilayer compression bandage for 2 days then stockings but duration not specified | Extended | 5000 units intravenous unfractionated heparin perioperatively then enoxaparin at prophylactic dose for bodyweight for 2 days at home | Asymptomatic | 5 days, 1, 3 months |
| Kim et al.^S101^ | 2021 | GSV, SSV, ASV | Prospective cohort study | UGFS | 2 - 5 | 60 | Not stated | 1 (1.7%) | Not stated | Eccentric compression for 24-48 hours, then 2 weeks compression at 20-30mmHg | Not stated | Not stated | Asymptomatic | 1 week, 3 and 6 months |
| Kim et al.^S102^ | 2016 | SSV | Retrospective cohort study | EVLA | Not stated | 62 | Not stated | 0 (0.0%) | 0 (0.0%) | Class II compression stocking for 3-7 days | Not stated | Not stated | Asymptomatic | 1 week, 1, 3, 6 and 12 months |
| King et al.^S103^ | 2015 | GSV | RCT | UGFS | 2 - 6 | 279 | 15 (5.4%) | 12 (4.3%) | 0 (0.0%) | Short stretch bandage with compression pads and a thigh length compression stocking 30-40mmHg for 48 hours, then compression stocking only in the day for 12 days | Not stated | Not stated | Asymptomatic | 1 week, if thrombus present repeat scan in 1 and 2 weeks |
| King et al.^S104^ | 2009 | GSV, SSV, ASV | Prospective cohort study | EVLA | 2 - 6 | 924 | Not stated | 1 (0.1%) | 0 (0.0%) | 30-40mmHg compression stocking for 36 hours then in the day for one week | Nil | Nil | Asymptomatic | 1 week, 1, 3, 6, 12 and 24 months |
| Knipp et al.^S105^ | 2008 | GSV | Retrospective cohort study | EVLA | 1 - 6 | 364 | 32 (9.9%) | 3 (0.8%) | 1 (0.3%) | Compression bandage for 48 hours then daily bandaging for 1 week, then thigh length compression stockings 30-40mmHg | Single shot | 2 risk factors: no heparin, 3-4 risk factors: 5000 units unfractionated heparin or 30mg enoxaparin, 5 or more risk factors received 1 week enoxaparin 30mg OD | Asymptomatic | days 0 to 30, 30 to 90, 90 to 180, 180 to 360 |
| Kontothanassis et al.^S106^ | 2009 | SSV | Prospective cohort study | EVLA | 2 - 6 | 204 | Not stated | 3 (1.5%) | 0 (0.0%) | Elastic bandage for 2 hours then class II compression stockings for 15 days | Extended | Reviparin 1750IU for 7 days | Asymptomatic | 1 week, 2 months, 1 year then yearly after |
| Koramaz et al.^S107^ | 2017 | GSV | Retrospective cohort study | Cyanoacrylate glue, EVLA | 2 - 5 | 189 | 3 (1.6%) | 0 (0.0%) | Not stated | The NBCA were mobilized after the treatment without any prescription of compression stockings. The laser group were prescribed class II compression stockings (thigh high) for 2 weeks after the treatment | Not stated | Not stated | Asymptomatic | Week 1 and 6, 12 months |
| Korkmaz et al.^S108^ | 2013 | GSV | Retrospective cohort study | RFA | 2 - 6 | 344 | 0 (0.0%) | 0 (0.0%) | Not stated | Not stated | Not stated | Not stated | Asymptomatic | 1 and 6 months |
| Kulkarni et al.^S109^ | 2013 | GSV | Prospective cohort study | UGFS | 2 - 6 | 776 | 16 (2.1%) | 1 (0.1%) | 1 (0.1%) | Compression bandaging for 2-4 days followed by class II compression stockings for 10-14 days | Nil | Nil | Asymptomatic | 2 weeks |
| Kulkarni et al.^S110^ | 2012 | GSV, SSV | Prospective cohort study | UGFS | 5 - 6 | 186 | 6 (3.2%) | 0 (0.0%) | 0 (0.0%) | Compression bandaging and full length class II compression stocking for two weeks being changed at one week; this was converted to a below-knee multilayer compression bandage at two weeks and a below-knee class II elastic stocking on healing. | Not stated | Not stated | Asymptomatic | 1 week |
| Kurihara et al.^S111^ | 2016 | GSV, SSV, ASV | Retrospective cohort study | EVLA, RFA | 2 - 6 | 7374 | 2270 (30.8%) | 35 (0.5%) | 1 (0.0%) | Elastic stocking over an elastic bandage for 24-48 hours then elastic stocking for 3 weeks. | Not stated | Not stated | Asymptomatic | 24-72 hours, 1 month, 1 year |
| Kurnicki et al.^S112^ | 2016 | GSV, SSV | Prospective cohort study | UGFS | 2 - 4 | 52 | Not stated | 0 (0.0%) | 0 (0.0%) | Class II compression stockings for 48 hours then during the day for 6 months, then class I compression stockings during the day for 6 months | Not stated | Not stated | Asymptomatic | 1 week, 1, 3, 6, and 12 months |
| Kutas et al.^S113^ | 2015 | GSV | Prospective cohort study | EVLA | 2, 3 | 100 | Not stated | 0 (0.0%) | Not stated | Elastic bandage for 24 hours then class II compression stockings for a month | Not stated | Not stated | Asymptomatic | 1 week and 1 month |
| Lakhwani et al.^S114^ | 2009 | GSV, SSV | Retrospective cohort study | EVLA | 2 - 6 | 117 | Not stated | 0 (0.0%) | Not stated | Not stated | Not stated | Not stated | Asymptomatic | 10 days, 1, 6, and 12 months |
| Lane et al.^S115^ | 2016 | GSV, SSV | RCT | MOCA, RFA | Median C4 | 170 | 1 (0.6%) | 1 (0.6%) | Not stated | Stockings for 2 weeks post-procedure | Single shot | Single prophylactic dose of LMWH after procedure | Asymptomatic | 1 and 6 months |
| Lattimer et al.^S116^ | 2012 | GSV | RCT | EVLA, UGFS | 2 - 6 | 100 | 5 (5.0%) | 6 (6.0%) | Not stated | 23–32 mmHg, graduated elastic compression stocking with a waist attachment was applied for 3 weeks | Not stated | Not stated | Asymptomatic | 3 weeks and 3 months |
| Lawrence et al.^S117^ | 2010 | GSV | Retrospective cohort study | RFA | 2 - 5 | 500 | 34 (6.8%) | 0 (0.0%) | Not stated | Compressive dressing | Nil | Nil, LMWH if level 4 or higher closure, level 3 or higher surgeons’ choice. LMWH used in those with mechanical heart valves or those with a history of pulmonary embolus | Asymptomatic | 48-72 hours |
| Lawson et al.^S118^ | 2018 | GSV | Prospective cohort study | EVLA, RFA | 2 - 5 | 311 | Not stated | 1 (0.3%) | Not stated | After both procedures, a stocking exerting 23 mm Hg of pressure at the ankle region was applied, patients were instructed to wear continuously for the first 24 hours. After 24 hours, advised to continue to wear the stocking on a voluntary basis during the day for 1 week | Single shot | All received thromboprophylaxis with a single dose of nadroparin 0.3 mL (9.5 IU anti-Xa/mL | Asymptomatic | 1 week, 6 weeks and 12, 24, 36, 48 and 60 months |
| Leopardi et al.^S119^ | 2019 | GSV | Prospective cohort study | EVLA | 1-4 | 59 | Not stated | 0 (0.0%) | Not stated | Compressive bandage then class II compression stocking for 1 week | Not stated | Not stated | Asymptomatic | 1 month and 1 year |
| Li et al.^S120^ | 2018 | GSV | RCT | UGFS | 2 - 4 | 94 | Not stated | 0 (0.0%) | 0 (0.0%) | Compression stockings, unspecified length of time | Not stated | Not stated | Symptomatic | 6 months |
| Liang et al.^S121^ | 2015 | GSV | RCT | EVLA | 3 - 5 | 60 | 3 (5.0%) | 0 (0.0%) | 1 (1.7%) | Compression bandage for 24 hours then graduated compression stockings 20-30mmHg for 3 months | Not stated | Not stated | Asymptomatic | Day 2 and 7, 1, 2, 3 and 6 months |
| Lin et al.^S122^ | 2012 | GSV | Retrospective cohort study | EVLA, RFA | 2 - 6 | 259 | 10 (3.9%) | 0 (0.0%) | 0 (0.0%) | Not stated | Not stated | Not stated | Asymptomatic | 5-7 days |
| Lobastov et al.^S123^ | 2020 | GSV, SSV, ASV, giacomini veins | Retrospective cohort study | UGFS | 2 - 5 | 196 | 57 (28.6%) | 3 (1.5%) | 0 (0.0%) | 23-32mmHg compression stockings for 72 hours, then daytime for 1 month | Nil | Nil, 9 received enoxaparin 40mg for 7days as had previous history VTE | Asymptomatic | 1-2 weeks, 1, 3, 6 and 12 months |
| Lugli et al.^S124^ | 2009 | GSV | RCT | EVLA | 2 - 6 | 100 | Not stated | 0 (0.0%) | 0 (0.0%) | Elastic stockings and eccentric compression for 1 week | Not stated | Not stated | Asymptomatic | 7 days |
| Lurie et al.^S125^ | 2013 | GSV | Prospective cohort study | RFA | 2 - 6 | 120 | 7 (5.8%) | 2 (1.7%) | Not stated | Compression bandages for 24 hours then 1 week of 20-30mmHg stockings | Not stated | Not stated | Asymptomatic | 36 hours and 1 month |
| Lurie et al.^S126^ | 2003 | GSV | RCT | RFA | 2 - 4 | 44 | Not stated | 0 (0.0%) | 0 (0.0%) | Stockings | Not stated | Not stated | Asymptomatic | 72 hours, 1 and 3 weeks, 4 months |
| Manfrini et al.^S127^ | 2000 | GSV, SSV, ASV | Prospective cohort study | RFA | 0 - 5 | 210 | Not stated | 3 (1.4%) | 1 (0.5%) | Compressive hosiery | Extended | Prophylactic LMWH before procedure and for 6 days after | Asymptomatic | 1, 6 weeks, 6 and 12 months |
| Mao et al.^S128^ | 2012 | GSV | Retrospective cohort study | EVLA | 1 - 6 | 138 | Not stated | 0 (0.0%) | 0 (0.0%) | Elastic bandage, three days later, a compression stocking (30mmHg) | Nil | Nil | Asymptomatic | 1 week and 6 months |
| Marsh et al.^S129^ | 2010 | GSV, SSV | Retrospective cohort study | EVLA, RFA | 0 - 6 | 2253 | 7 (0.3%) | 8 (0.4%) | 1 (0.0%) | 20–30 mmHg graduated compression stockings to wear 24 h a day for at least 3 days | Single shot | Single dose of prophylactic LMWH | Asymptomatic | 2 weeks |
| Memetoglu et al.^S130^ | 2012 | GSV | Prospective cohort study | EVLA | 2 - 4 | 90 | Not stated | 1 (1.1%) | 0 (0.0%) | Elastic bandages for three days and class II (30–40 mmHg) stockings for at least one month | Nil | Nil | Asymptomatic | 1 week, 1, 3, 6, and 12 months |
| Memetoglu et al.^S131^ | 2020 | SSV | Prospective cohort study | UGFS | 2 - 5 | 30 | Not stated | 0 (0.0%) | 0 (0.0%) | Elastic bandaged for 48 hours then class II stockings in the day for 1 month | Not stated | Not stated | Asymptomatic | 1 week and then at last follow up (average 7 months) |
| Mendes-Pinto et al.^S132^ | 2015 | GSV | RCT | EVLA | 3 | 77 | Not stated | 0 (0.0%) | 0 (0.0%) | Knee high stocking with 20-30mmHg compression for 30 days | Single shot | Enoxaparin 40mg 6 hours after anaesthetic | Asymptomatic | 7 and 30 days, 3, 6 and 12 months |
| Merchant et al.^S133^ | 2005 | GSV, SSV | Prospective cohort study | RFA | 0 - 6 | 1006 | Not stated | 11 (1.1%) | 1 (0.1%) | Not stated | Not stated | Not stated | Asymptomatic | 1 week, 6 months, 1 year, and yearly to 5 years |
| Merchant et al.^S134^ | 2002 | GSV, SSV | Prospective cohort study | RFA | 0 - 6 | 286 | Not stated | 3 (1.0%) | 1 (0.3%) | Not stated | Not stated | Not stated | Asymptomatic | 1 week, 6, 12, 14 and 24 months |
| Mese et al.^S135^ | 2015 | GSV | RCT | EVLA, RFA | Mean C 3.4 | 120 | Not stated | 0 (0.0%) | 0 (0.0%) | An elastic bandage was applied to the leg receiving the procedure for 2 days after which compression stockings were recommended for 3 months | Not stated | Not stated | Asymptomatic | 1 week, 1, 3 and 6 months |
| Min et al.^S136^ | 2003 | GSV | Prospective cohort study | EVLA | Not stated | 423 | Not stated | 0 (0.0%) | Not stated | Class II compression stockings for at least one week | Not stated | Not stated | Asymptomatic | 1 week, 1, 3, 6, 9 and 12 months |
| Mohamed et al.^S137^ | 2021 | GSV, SSV, ASV | RCT | EVLA, MOCA | 2 - 6 | 150 | Not stated | 1 (0.7%) | 0 (0.0%) | Elastic compression bandage to groin for 24 hours then class I graduated compression stockings for 6 days; C6 given 4-layer compression | Nil | Nil | Asymptomatic | 1, 6, 26 and 52 weeks |
| Mohammadi-Tofigh et al.^S138^ | 2020 | GSV | RCT | EVLA, RFA | 2 - 6 | 1090 | Not stated | 3 (0.3%) | Not stated | Compression bandage for 48 hours then class II stocking for 4-8 weeks | Not stated | Not stated | Asymptomatic | Day 7, then 3, 6, and 12 months |
| Morrison et al.^S139^ | 2015 | GSV | RCT | Cyanoacrylate glue, RFA | 2 - 4 | 222 | 0 (0.0%) | 0 (0.0%) | 0 (0.0%) | Post-treatment stockings were worn by both subjects because the RFA instructions for use require compression | Not stated | Not stated | Asymptomatic | Day 3, months 1 and 3 |
| Moul et al.^S140^ | 2014 | GSV, SSV | Retrospective cohort study | EVLA | 2 - 6 | 1171 | Not stated | 0 (0.0%) | 0 (0.0%) | Support stockings | Not stated | Not stated | Asymptomatic | 1 week, and 1, 6, 12, and 24 months |
| Mozes et al.^S141^ | 2005 | GSV | Retrospective cohort study | EVLA | 2 - 6 | 32 | 3 (9.4%) | 0 (0.0%) | Not stated | Not stated | Not stated | Not stated | Asymptomatic | 1 week |
| Murli et al.^S142^ | 2013 | GSV, SSV | Retrospective cohort study | EVLA | 5 - 6 | 145 | Not stated | 2 (1.4%) | Not stated | Multi-layered bandaging or graduated compression stockings | Not stated | Not stated | Asymptomatic | 1 month, 1 and 2 years |
| Myers et al.^S143^ | 2007 | GSV, SSV | Prospective cohort study | UGFS | 2 - 6 | 807 | Not stated | 16 (2.0%) | 0 (0.0%) | Two layer bandage or class II stocking for 3 days, then compression stocking to be worn in the day | Not stated | Not stated | Asymptomatic | 3-5 days then 6 weeks, 6, 12, 18 and 24 months |
| Myers et al.^S144^ | 2008 | GSV, SSV, ASV | Retrospective cohort study | UGFS | 2 - 6 | 1931 | Not stated | 28 (1.5%) | 1 (0.1%) | Not stated | Single shot | Enoxaparin 40mg one shot | Asymptomatic | Post-op and day 3 -7 |
| Nael et al.^S145^ | 2010 | GSV, SSV | Retrospective cohort study | UGFS | 2 - 6 | 166 | Not stated | 5 (3.0%) | 0 (0.0%) | 30-40mmHg compression stocking and/or short stretch bandage | Not stated | Not stated | Asymptomatic | Post-procedural then 1 week |
| Navarro et al.^S146^ | 2001 | GSV | Prospective cohort study | EVLA | Not stated | 33 | Not stated | 0 (0.0%) | 0 (0.0%) | Class II compression stockings for 7 days | Not stated | Not stated | Asymptomatic | 1 day, 1 week and then monthly |
| Nemoto et al.^S147^ | 2019 | GSV, SSV, ASV | Retrospective cohort study | EVLA | 2 - 6 | 43,203 | 375 (0.9%) | 24 (0.1%) | 3 (0.0%) | Not stated | Not stated | Not stated | Asymptomatic | 72 hours and 1-3 months |
| Nishibe et al.^S148^ | 2017 | GSV, SSV | Prospective cohort study | RFA | 2 - 4 | 91 | 0 (0.0%) | 0 (0.0%) | Not stated | Graduated compression bandage for 24 hours then graduated stocking | Not stated | Not stated | Asymptomatic | 1 week, 1 month and 4 months |
| Nordon et al.^S149^ | 2011 | GSV | RCT | EVLA, RFA | 2 - 6 | 157 | Not stated | 0 (0.0%) | 0 (0.0%) | Compression bandage for 24 hours then compression hosiery for 2 weeks minimum | Nil | Nil | Asymptomatic | 1 week and 3 months |
| Novotny et al.^S150^ | 2018 | GSV, SSV | Prospective cohort study | Cyanoacrylate glue | 2 - 6 | 49 | 2 (4.0%) | 0 (0.0%) | 1 (2.0%) | Compression stocking for 24 hours | Nil | Nil | Asymptomatic | 1 and 6 days, 6 weeks, 6 months, 1 and 2 years |
| Nwaejike et al.^S151^ | 2010 | GSV, SSV | Prospective cohort study | EVLA | 2 - 5 | 77 | Not stated | 0 (0.0%) | 1 (1.3%) | Class II graduated compression stockings for 1 week then only in day for 1 week | Not stated | Not stated | Symptomatic | Median 18 months |
| O'Hare et al. ^S152^ | 2008 | GSV, SSV | Prospective cohort study | UGFS | 1 - 6 | 165 | Not stated | 1 (0.6%) | Not stated | 14-17mmHg compression stocking over bandages, removed after 5 days then bandage replaced for 2 weeks | Not stated | Not stated | Asymptomatic | 2 weeks and 6 months |
| Obi et al.^S153^ | 2015 | GSV, SSV | Prospective cohort study | RFA | 2 - 6 | 399 | 10 (2.5%) | 6 (1.5%) | 0 (0.0%) | Compressed for at least 2 weeks | Single/ Extended | 5000 IU heparin except in 42 operated under local, with a 2006 Caprini risk score ≥ 8 also received a standard protocol of daily enoxaparin prophylaxis (40 mg) for one week postoperatively, starting the day after the procedure | Asymptomatic | 7 days, 3 and 12 months |
| Ontas et al.^S154^ | 2019 | GSV | Prospective cohort study | EVLA, RFA | 2 - 4 | 50 | Not stated | 0 (0.0%) | Not stated | Class II compression stockings for 3 months | Not stated | Not stated | Symptomatic | 6 months |
| Onwudike et al.^S155^ | 2020 | GSV, SSV, ASV | RCT | RFA | 2 - 6 | 94 | 2 (2.1%) | 2 (2.1%) | Not stated | 48 had 23-32mmHg compression stocking for one week all day and night then just all day for another week | Nil | Nil | Asymptomatic | 2 and 12-14 weeks |
| Pannier et al.^S156^ | 2009 | GSV, SSV | Prospective cohort study | EVLA | 2 - 6 | 94 | Not stated | 0 (0.0%) | 0 (0.0%) | Eccentric compression and 23–32 mmHg compression stocking were applied for 24 hour | Extended | Prophylactic dose LMWH for 7 days | Asymptomatic | 1 and 10 days, 1, 6 and 12 months |
| Pannier et al.^S157^ | 2008 | GSV, SSV | Prospective cohort study | EVLA | 2 - 6 | 65 | Not stated | 0 (0.0%) | 0 (0.0%) | Eccentric compression for 24 hours then 30mmHg stocking applied for 3 weeks | Extended | Prophylactic dose LMWH for 10 days | Asymptomatic | 1 and 8 days |
| Pannier et al.^S158^ | 2011 | GSV | Prospective cohort study | EVLA | 2 - 5 | 50 | Not stated | 0 (0.0%) | 0 (0.0%) | Eccentric compression for 24 hours then 30mmHg stocking applied for 1 month | Extended | Prophylactic dose LMWH for 7 days | Asymptomatic | 1, 10 and 30 days, 6 months |
| Park et al.^S159^ | 2008 | SSV | Prospective cohort study | EVLA | 1 - 4 | 344 | Not stated | 0 (0.0%) | 0 (0.0%) | Class II compression for 2 days then compression in the day for 3-4 weeks | Not stated | Not stated | Asymptomatic | 1 week, 1, 3, 6, 12 and 24 months |
| Parsi et al.^S160^ | 2020 | GSV, SSV | Prospective cohort study | EVLA, UGFS | Not stated | 1325 | 1 (0.1%) | 7 (0.5%) | 0 (0.0%) | Class II compression stockings for 7 days | Not stated | Not stated | Asymptomatic | 1 and 6 weeks, 12 months then annually |
| Perkowski et al.^S161^ | 2004 | GSV, SSV, ASV | Prospective cohort study | EVLA | 2 - 6 | 165 | Not stated | 0 (0.0%) | Not stated | Three layer compression wrap for 2 days then compression stockings during the day for 2 weeks | Not stated | Not stated | Asymptomatic | 1 week and 1 year |
| Perrins et al. ^S162^ | 2013 | GSV | Retrospective cohort study | RFA | 0 - 6 | 171 | 1 (0.6%) | 0 (0.0%) | Not stated | Not stated | Not stated | Not stated | Asymptomatic | 3 days and 3 months |
| Pleister et al.^S163^ | 2008 | GSV | Prospective cohort study | EVLA | 2 - 6 | 50 | Not stated | 0 (0.0%) | 0 (0.0%) | Not stated | Not stated | Not stated | Asymptomatic | 24 hours and 3 months |
| Poschinger-Figueiredo et al.^S164^ | 2021 | GSV | Prospective cohort study | RFA | 3 - 6 | 30 | Not stated | 0 (0.0%) | 0 (0.0%) | Compression bandage for 48 hours | Not stated | Not stated | Asymptomatic | 1 week, 6 months, 1 and 3 years |
| Proebestle et al.^S165^ | 2008 | GSV | Prospective cohort study | RFA | 2 - 6 | 194 | Not stated | 0 (0.0%) | Not stated | Eccentric compression bandage for 24-72 hours then 30mmHg compression stockings for 8 days | Not stated | Not stated | Asymptomatic | 3 days, 3 and 6 months |
| Proebestle et al.^S166^ | 2003 | SSV | Prospective cohort study | EVLA | 2 - 6 | 33 | Not stated | 1 (3.0%) | Not stated | Eccentric compression bandage for 24-48 hours then class II compression stockings for 8 days | Extended | 2500 IU dalteparin for 8 days | Asymptomatic | Day 1 and 1, 3, 6 and 12 months |
| Proebestle et al.^S167^ | 2006 | GSV | Prospective cohort study | EVLA | 2 - 6 | 203 | Not stated | 0 (0.0%) | 0 (0.0%) | Eccentric compression bandage was applied over the course of the treated vein for 24 hours. Additionally, the patient had to wear graduated compression stockings (30 mm Hg) continuously day and night for 8 days | Extended | LMWH (2500 IU dalteparin subcutaneously) once daily. | Asymptomatic | 1 day, 1, 3, 6 and 12 months |
| Puggioni et al.^S168^ | 2009 | GSV | Retrospective cohort study | RFA | 2 - 6 | 293 | 24 (8.2%) | 14 (4.8%) | 0 (0.0%) | Compressive bandage for 48 hours | Nil | Nil | Asymptomatic | 5.8 days on average then 1-2 and 6 months |
| Rasmussen et al.^S169^ | 2011 | GSV | RCT | EVLA, RFA, UGFS | 2 - 6 | 424 | Not stated | 1 (0.2%) | 1 (0.2%) | Compression bandage for 48 hours, then 30mmHg compression stocking for UGFS or 20mmHg stocking for other for 2 weeks | Not stated | Not stated | Asymptomatic | 3 days, 1 month and 1 year |
| Rasmussen et al.^S170^ | 2007 | GSV | RCT | EVLA | 2 - 6 | 121 | 1 (0.8%) | 0 (0.0%) | Not stated | Compressive bandage for 48 hours then class I compression stockings for 2 weeks | Not stated | Not stated | Asymptomatic | 12 days, 1, 3, and 6 months |
| Rass et al.^S171^ | 2012 | GSV | RCT | EVLA | 2 - 5 | 185 | 2 (1.1%) | 1 (0.5%) | Not stated | Compression bandage for 24 hours then class II thigh compression stockings for 4 weeks | Extended | Tinzaparin 42.2mg OD for 6 days | Asymptomatic | 1 week, 3 months, 1 and 2 years |
| Rathod et al.^S172^ | 2010 | GSV, SSV | Prospective cohort study | EVLA | 2 - 6 | 72 | 0 (0.0%) | 0 (0.0%) | 0 (0.0%) | Class II compression stocking | Not stated | Not stated | Asymptomatic | 2 days, 1, 6, and 12 months |
| Ravi et al.^S173^ | 2006 | GSV, SSV | Retrospective cohort study | EVLA, RFA | 2 - 6 | 981 | 0 (0.0%) | 0 (0.0%) | 1 (0.1%) | Not stated | Nil | Nil, LMWH was given to 3 as suggested by their haematologist due to hypercoagulable disease. | Asymptomatic | 2 weeks then 6 and 12 months |
| Reich-Schupke et al.^S174^ | 2013 | Truncal but not specified | Retrospective cohort study | UGFS | 2 - 6 | 54 | 0 (0.0%) | 0 (0.0%) | Not stated | 23-32mmHg compression for 3 weeks | Extended | LMWH prophylactic dose OD 3 days | Asymptomatic | Every 2-3 weeks |
| Rustempasic et al.^S175^ | 2014 | GSV | Prospective cohort study | EVLA | 2 | 61 | Not stated | 0 (0.0%) | Not stated | Compression bandages for 7 days | Not stated | Not stated | Symptomatic | No duplex post-procedure |
| Ryer et al.^S176^ | 2016 | GSV | Retrospective cohort study | EVLA, RFA | 1 - 6 | 842 | 43 (5.1%) | 4 (0.5%) | 1 (0.1%) | Not stated | Not stated | Not stated | Asymptomatic | 1 day then 1 week |
| Samuel et al.^S177^ | 2013 | SSV | Prospective cohort study | EVLA | 2 - 5 | 76 | Not stated | 0 (0.0%) | 0 (0.0%) | Elastic compression bandage for 1 week then class II graduated compression stockings for 5 weeks | Not stated | Not stated | Asymptomatic | 1, 6, 12, 52, 104 weeks and 5 years |
| Schwarz et al.^S178^ | 2010 | GSV | Prospective cohort study | EVLA | 2 - 6 | 286 | 1 (0.3%) | 2 (0.7%) | 1 (0.3%) | Class II compression stocking for 1 week, then during the day for a further 3 weeks | Extended | Enoxaparin 40mg OD for 5 days | Asymptomatic | 1 week, 1 month, 3 months |
| Sermsathenesawadi et al.^S179^ | 2016 | GSV | Retrospective cohort study | RFA | 2 - 6 | 82 | 10 (12.2%) | 0 (0.0%) | 0 (0.0%) | Compression bandage for 24 hours then graduated compression stocking for 1 week | Nil | Nil | Asymptomatic | 1 week, 1 and 3 months |
| Sevil et al.^S180^ | 2020 | GSV | Retrospective cohort study | RFA | 2 - 5 | 100 | Not stated | 1 (1.0%) | 0 (0.0%) | Elastic bandage for 2-5 days then compression socks 20-30mmHg for 6 months | Not stated | Not stated | Asymptomatic | 1 day, 1 week, 1, 6, 12 and 24 months |
| Shadid et al. ^S181^ | 2012 | GSV | RCT | UGFS | 2 - 5 | 230 | Not stated | 1 (0.4%) | 1 (0.4%) | Compression was applied with a foam pad over the treated area and an antiembolism stocking for 1 week, day and night. A class II elastic stocking was prescribed during the day for 6 weeks | Not stated | Not stated | Symptomatic | 3 months, 1 and 2 years |
| Shoab et al.^S182^ | 2016 | GSV | Prospective cohort study | EVLA | 2 - 6 | 131 | 1 (0.8%) | 0 (0.0%) | Not stated | Compression with class 2 stockings on the table, worn for 1 week continuously. Stockings advised in daytime for 1 week after. | Nil | Nil | Symptomatic | 6 weeks |
| Sharif et al.^S183^ | 2006 | GSV | Prospective cohort study | EVLA | Not stated | 136 | Not stated | 0 (0.0%) | Not stated | A full-length compression stocking (class II support hosiery) was then applied for 7 days. | Not stated | Not stated | Asymptomatic | 1 week, 3 and 12 months |
| Shepherd et al.^S184^ | 2010 | GSV, SSV | RCT | EVLA, RFA | 1 - 6 | 131 | Not stated | 0 (0.0%) | 1 (0.8%) | Thromboembolic deterrent stocking for 1 week post-op | Single shot | 5000 units subcutaneous unfractionated heparin | Symptomatic | Duplex in theatre post-op then no further unless DVT symptoms |
| Shutze et al.^S185^ | 2016 | GSV, SSV | Retrospective cohort study | EVLA | 2 - 6 | 1109 | 76 (6.9%) | 0 (0.0%) | Not stated | Compression bandages or stockings post-op | Not stated | Not stated | Asymptomatic | Post-procedural then 1 week |
| Smith et al.^S186^ | 2006 | GSV, SSV | Retrospective cohort study | UGFS | 1 - 6 | 808 | 2 (0.2%) | 11 (1.4%) | Not stated | Short stretch compression bandaging and class 2 compression stocking for 3-14 days | Nil | Nil, a small number with duplex ultrasound evidence of post-thrombotic deep vein damage was treated. All received a 5 day course of prophylactic LMWH | Asymptomatic | 2 weeks then 6 months |
| Spinedi et al.^S187^ | 2020 | GSV | Prospective cohort study | EVLA | 2 - 5 | 113 | 2 (1.8%) | 1 (0.9%) | 0 (0.0%) | Eccentric compression bandage then full length class II compression stocking | Extended | Rivaroxaban 10mg OD 5 days | Asymptomatic | 1 and 10 days, 6 weeks |
| Spreafico et al.^S188^ | 2011 | GSV, SSV | Retrospective cohort study | EVLA | 1 - 6 | 1020 | 5 (0.5%) | 1 (0.1%) | 0 (0.0%) | Not stated | Not stated | Not stated | Asymptomatic | 1 week or 1 month then 1 year |
| Starodubstev et al.^S189^ | 2017 | GSV | Prospective cohort study | EVLA | 4 - 6 | 476 | Not stated | 0 (0.0%) | 0 (0.0%) | Compression therapy for 2 months or until ulcer healing | Not stated | Not stated | Asymptomatic | 2 days, 2 weeks, 2 and 6 months, 1, 2, and 3 years |
| Stone et al.^S190^ | 2006 | GSV | Prospective cohort study | RFA | Not stated | 51 | 2 (3.9%) | 1 (2.0%) | Not stated | Compression bandage for 48 hours then compression stockings for 5 days | Not stated | Not stated | Asymptomatic | 5 days |
| Sufian et al.^S191^ | 2017 | GSV, SSV | Case control | EVLA, RFA | 1 - 6 | 753 | 8 (1.1%) | 6 (0.8%) | Not stated | 30-40mmHg compression stockings to proximal thigh for 1 week | Extended | Chronic antithrombotic (warfarin n=292, rivaroxaban n=49, dabigatran n=36, apixaban n=1) ± aspirin (n=44) ± clopidogrel (n=4) | Asymptomatic | 3 days then 1 month |
| Sufian et al.^S192^ | 2013 | GSV, SSV | Prospective cohort study | RFA | 1 - 6 | 6707 | 201 (3.0%) | 12 (0.2%) | 2 (0.0%) | Elastic bandage for 24 hours | Nil | Nil | Asymptomatic | 2-3 days, 1, 6 and 12 months |
| Sufian et al.^S193^ | 2015 | GSV | Prospective cohort study | RFA | Mean 2.6 | 409 | 11 (2.7%) | 0 (0.0%) | 0 (0.0%) | Elastic bandage for 24 hours | Nil | Nil | Asymptomatic | 3-5 days and 1 month |
| Sutton et al.^S194^ | 2012 | GSV, SSV | Retrospective cohort study | EVLA, UGFS | Not stated | 5828 | Not stated | 18 (0.3%) | 3 (0.1%) | Not stated | Not stated | Not stated | Symptomatic | Nil |
| Sydnor et al.^S195^ | 2016 | GSV | RCT | EVLA, RFA | 2 - 6 | 200 | 0 (0.0%) | 0 (0.0%) | Not stated | Compression stockings for 24 hours then during day for 14 days | Not stated | Not stated | Asymptomatic | 7 days, 6 weeks and 6 months |
| Takahashi et al.^S196^ | 2017 | GSV, SSV | Retrospective cohort study | EVLA | 2 - 6 | 1136 | 10 (0.9%) | 1 (0.1%) | 0 (0.0%) | Elastic bandage for 24 hours then elastic stocking for 1 week | Extended | Long-term antithrombotics | Asymptomatic | Day 1, week 1 then 2-5 months |
| Tamura et al.^S197^ | 2017 | GSV, SSV | Retrospective cohort study | RFA | 2 - 4 | 100 | 5 (5.0%) | 0 (0.0%) | Not stated | Not stated | Not stated | Not stated | Asymptomatic | 1 day, 1 week, 1, 3, 6 and 12 months |
| Tang et al.^S198^ | 2017 | GSV, SSV | Prospective cohort study | MOCA | 2 - 6 | 300 | Not stated | 0 (0.0%) | Not stated | Compression stocking (23 mmHg) graduated pressure from ankle to upper thigh for three weeks | Nil | Nil | Symptomatic | 2 months |
| Tawfik et al.^S199^ | 2020 | GSV, SSV, ASV | RCT | EVLA, MOCA | 2 - 4 | 100 | 0 (0.0%) | 0 (0.0%) | 0 (0.0%) | Not stated | Single shot | Single dose of enoxaparin (20 or 40 mg according to patient's weight) just before the procedure | Asymptomatic | 1 week, 1, 6 and 12 months |
| Theivacumar et al.^S200^ | 2007 | SSV | Prospective cohort study | EVLA | 2- 6 | 68 | Not stated | 0 (0.0%) | Not stated | Following treatment a non-stretch compression bandage was applied to the limb for 1 week followed by a class 2 support stocking for a further week | Not stated | Not stated | Symptomatic | 6 weeks, 3 months, 6 months |
| Theivacumar et al.^S201^ | 2009 | GSV | Retrospective cohort study | EVLA | 2 - 6 | 44 | Not stated | 0 (0.0%) | Not stated | Not stated | Extended | Warfarin | Symptomatic | 6, 12 then 52 weeks |
| Timperman et al.^S202^ | 2004 | GSV, SSV, ASV | Prospective cohort study | EVLA | Not stated | 87 | 1 (1.1%) | 0 (0.0%) | Not stated | Graduated compression hose for 1 week then for 2-3 weeks in the day | Not stated | Not stated | Asymptomatic | 1 week |
| Todd et al.^S203^ | 2014 | GSV, ASV | RCT | UGFS | 2-6 | 232 | Not stated | 13 (5.6%) | 0 (0.0%) | 30-40mmHg compression stockings and compression bandages for 48 hours then stockings only for 12 more days | Not stated | Not stated | Asymptomatic | 1 week, 8 weeks, 1 year (if thrombus detected at 1 week then scanned weekly until clot stabilized or dissolved) |
| Tolva et al.^S204^ | 2013 | GSV | Prospective cohort study | RFA | 2 - 6 | 398 | 1 (0.3%) | 0 (0.0%) | Not stated | All were required to wear class I compression stockings for 3 weeks | Extended | 4000 IU LMWH for 4 days | Asymptomatic | 7–10 days, 1, 3 and 6 months |
| Toniolo et al.^S205^ | 2018 | GSV, SSV | Prospective cohort study | UGFS | 2 - 6 | 290 | Not stated | 15 (5.2%) | Not stated | Stockings for 6 weeks with bandages initially | Not stated | Not stated | Symptomatic | 6 weeks, 1 year |
| Trip-Hoving et al.^S206^ | 2009 | SSV | Retrospective cohort study | EVLA | 0 - 6 | 49 | Not stated | 1 (2.0%) | Not stated | Compression stockings (30-40mmHg) for 7 days | Not stated | Not stated | Symptomatic | 6 weeks |
| Uthoff et al.^S207^ | 2017 | GSV, SSV, ASV | Retrospective cohort study | EVLA | 2 - 6 | 310 | 19 (6.1%) | 0 (0.0%) | Not stated | Eccentric compression and full-length class II stockings for 2-3 weeks during the day | Extended | Rivaroxaban 10mg OD for 5 or 10 days | Asymptomatic | 1 day, 4-6 weeks |
| Vahaaho et al.^S208^ | 2019 | GSV | RCT | EVLA, MOCA, RFA | 2 - 4 | 125 | Not stated | 0 (0.0%) | Not stated | A class 2 compression stocking was applied, with the instruction to wear the stocking continuously for 48 h, then during the day until 2 weeks after the operation. Wound care and aftercare were identical in the three groups | Not stated | Not stated | Asymptomatic | 1 month |
| Van Eereken et al.^S209^ | 2012 | GSV | Prospective cohort study | MOCA, RFA | 1 - 6 | 68 | Not stated | 0 (0.0%) | 0 (0.0%) | Compression stocking 30-40 mmHg for 2 weeks | Not stated | Not stated | Symptomatic | DUS not reported |
| Vasquez et al.^S210^ | 2007 | GSV, SSV, ASV | Prospective cohort study | RFA | 2 - 6 | 499 | Not stated | 1 (0.2%) | Not stated | Elastic bandage for 24 hours then compression recommended indefinitely | Nil | Nil | Asymptomatic | 4 days, 4 weeks, 4 months then every 6 months for |
| Von Hodenberg et al.^S211^ | 2015 | GSV, SSV | Prospective cohort study | EVLA | 2 - 6 | 308 | Not stated | 0 (0.0%) | 0 (0.0%) | Compression with class 2 stockings (30-40mmHg) for 24 hrs/day for 7 days then during the day for further 3 weeks | Extended | Enoxaparin 40mg on the day and then for 5 days | Asymptomatic | 1 week, 1 month, 3 months, 1 year |
| Vuylsteke et al.^S212^ | 2010 | GSV | Prospective cohort study | EVLA | 2 - 3 | 129 | Not stated | 0 (0.0%) | Not stated | Compression stockings (class 2) were applied for three weeks postoperatively | Extended | Enoxaparin 20mg 10 days | Asymptomatic | 1 month |
| Vuylsteke et al.^S213^ | 2011 | GSV | RCT | EVLA | 2 - 6 | 180 | Not stated | 0 (0.0%) | Not stated | Compression stockings (class 2) were applied for three weeks postoperatively. | Extended | All received prophylactic LMWH (Enoxaparin 40 mg) for 10 days. | Asymptomatic | 1 and 6 months |
| Watanabe et al.^S214^ | 2021 | GSV, SSV | RCT | EVLA | 1 - 6 | 467 | 2 (0.4%) | 0 (0.0%) | 0 (0.0%) | Elastic compression stockings for 24 hours then during the day for 1 month | Nil | Nil | Asymptomatic | 1 day, 1 week, 1 month, 1 year |
| Weiss et al.^S215^ | 2002 | GSV | Retrospective cohort study | RFA | Not stated | 120 | Not stated | 0 (0.0%) | 0 (0.0%) | Short stretch bandage for 24 hours then 30-40mmHg graduated compression stockings for 2 weeks | Not stated | Not stated | Asymptomatic | 1 week, 6 weeks, 6 months, 1 year, and 2 years |
| Welch et al.^S216^ | 2006 | GSV | Retrospective cohort study | RFA | 2 - 6 | 146 | Not stated | 0 (0.0%) | Not stated | Compression stockings for 72 hrs | Not stated | Not stated | Asymptomatic | 1 week, then 2-3 months |
| Yamamoto et al.^S217^ | 2021 | GSV, SSV | Retrospective cohort study | RFA | 2 - 6 | 1334 | 4 (0.3%) | 0 (0.0%) | 0 (0.0%) | Compression class II stockings all day for 7 days and then for 2 more weeks if oedema occurred | Not stated | Not stated | Asymptomatic | 1 - 5 days, 1 month, 3 months |
| Yang et al.^S218^ | 2018 | GSV, SSV, ASV | Prospective cohort study | Cyanoacrylate glue, RFA | 2 - 5 | 335 | 2 (0.6%) | 0 (0.0%) | Not stated | Those treated with RFA were all instructed to wear compression stockings until at least the first follow-up visit | Not stated | Not stated | Asymptomatic | 1 and 8 weeks |
| Yilmaz et al.^S219^ | 2011 | GSV, SSV | Retrospective cohort study | EVLA | 1 - 6 | 504 | Not stated | 5 (1.0%) | Not stated | Used but duration unclear | Not stated | Not stated | Asymptomatic | 1 month, 6 months, 12 months |
| Zhu et al.^S220^ | 2019 | GSV, SSV | Retrospective cohort study | UGFS | 6 | 35 | Not stated | 0 (0.0%) | 0 (0.0%) | Used but duration unclear | Not stated | Not stated | Asymptomatic | 2 weeks and 12 months |
| Zuniga et al.^S221^ | 2012 | GSV | Prospective cohort study | RFA | 1 - 6 | 667 | Not stated | 11 (1.6%) | Not stated | Not stated | Not stated | Not stated | Asymptomatic | 1 week, 3 months, 6 months, 1 year |

**Supplemental Digital Content 4 – Cochrane risk of bias assessment for randomised trials.**

| **Author** | **Year** | **Study Design** | **Randomisation** | **Allocation concealment** | **Blinding of participants and personelle** | **Blinding of outcome assessment** | **Attrition** | **Reporting** | **Overall** |
| --- | --- | --- | --- | --- | --- | --- | --- | --- | --- |
| Shepherd et al.^S183^ | 2010 | RCT | Low risk - internet randomisation | Low risk - blinded patients | Low risk - not possible to blind participants and personelle to treatment group but unlikely to influence performance of the trial | Low risk - assessors not blinded but outcome of DVT unlikely to be influenced by knowledge of the intervention as measured with objective means | Low risk - intention to treat | Low risk - study had a protocol | Low risk |
| Campos Gomes et al. ^S30^ | 2020 | RCT | Low risk - random computer numbers | Unclear risk of bias - recorded in patient chart and reviewed with patient on day of procedure | Low risk - not possible to blind participants and personelle to treatment group but unlikely to influence performance of the trial | Low risk - assessors not blinded but outcome of DVT unlikely to be influenced by knowledge of the intervention as measured with objective means | Low risk - no statistical difference in dropouts between groups | Low risk - registered trial | Unclear risk |
| Eroglu et al.^S63^ | 2018 | RCT | Low risk - blocked randomisation | Low risk - sealed envelopes | Low risk - not possible to blind participants and personelle to treatment group but unlikely to influence performance of the trial | Low risk - "DUS was performed by a radiologist in a blinded manner" | High risk - Seven (4%) of the patients undergoing NBCA, 26 (14.8%) of those undergoing RFA and 36 (20.5%) of those undergoing EVLA were lost during two year follow up. Four hundred and fifty six patients completed 2 years of follow up. | High risk of bias | High risk |
| Gohel et al. ^S77^ | 2018 | RCT | low risk (Randomization sequences for each recruitment center were created with the use of randomly permuted blocks with two block sizes) | low risk (once elegibility was confirmed, patients were randomly assigned to each block in a 1:1 ratio by a computer) | Low risk - not possible to blind participants and personelle to treatment group but unlikely to influence performance of the trial | Unclear risk - not specified in the paper | low risk (intention to treat analysis used, similar attrition rates in both groups) | low risk (study protol published before the trial http://www.controlled-trials.com/ISRCTN02335796 and all pre-specified outcomes reported) | Unclear risk |
| Hamel-Desnos et al.^S79^ | 2009 | RCT | Unclear - Each treatment was assigned to the patients according to the randomization list | Unclear - Each treatment was assigned to the patients according to the randomization list | Unclear risk - not specified in the paper | Unclear risk - not specified in the paper | Unclear - no mention of follow up | High risk - no protocol | High risk |
| Holewijn et al.^S85^ | 2018 | RCT | Low risk - online randomisation | Unclear - no mention of whether the allocation was concealed | Unclear risk - not specified in the paper | Low risk - blinded vascular scientists | High risk - Attrition rate >10% and differential between the two groups over 2 years | Low risk - registered protocol | High risk |
| Gibson et al.^S71^ | 2017 | RCT | Low risk - computer generated | Low risk - Sealed envelopes | Low risk - Patients and independent observers remained blinded through Week 12. | Unclear - assesors blinded for some things but DUS not specified | Low risk - all completed blinded part of bias | High risk - registered trial but didn't include all endpoints in protocol | High risk |
| King et al.^S103^ | 2015 | RCT | Low risk - | Unclear - allocation concealment not specified | Low risk - Drapes or screening were used to obscure the patient's view of the treatment procedure; clinicians attempted to be blinded but the foam placebo was distinguishable | Low risk - assessors for all primary and secondary endpoints were completely blinded | Low risk - low attrition in each group | High risk - registered trial but didn't include all endpoints in protocol | High risk |
| Todd et al.^S202^ | 2014 | RCT | Low risk - automated interactive voice recognition | Unclear - allocation concealment not specified | Low risk - drug administered double blinded but placebo single blinded | Low risk - outcome asseors blinded to treatment allocation and timepoint which reviewed the images | Low risk - low attrition rates across groups | High risk - registered trial but didn't include all endpoints in protocol | High risk |
| Lane et al.^S115^ | 2016 | RCT | Low risk - computerised randomisation software | Unclear - allocation concealment not specified | Low risk - knowledge of intervention but unlikely to affect outcome | Low risk - Technical success was also assessed at one month and six months with validated, blinded venous duplex ultrasound scanning | High risk - 30% loss to follow up in each group | Low risk - registered protocol | High risk |
| Li et al.^S120^ | 2018 | Prospective cohort study | Low risk - Randomization was performed using a sealed envelope, and the treated patients were asked to pick up a sealed envelope blindly. | Low risk - Randomization was performed using a sealed envelope, and the treated patients were asked to pick up a sealed envelope blindly. | Unclear - not stated | Unclear - not stated | Unclear - attrition not stated | High risk - no protocol | High risk |
| Liang et al.^S121^ | 2015 | RCT | Low risk - a computer-generated randomization list | Unclear - allocation concealment not specified | Unclear - not stated | Unclear - not stated | Low risk - no attrition | High risk - no protocol | High risk |
| Lurie et al.^S125^ | 2003 | RCT | Low risk - Randomization was allocated via internet, The internet site was audited regularly to detect redundant randomization activity | Low risk - Randomization was allocated via internet, The internet site was audited regularly to detect redundant randomization activity | Unclear - not stated | Unclear - not stated | Low risk - minimal attrition (1 in RFA 2 in surgery) | High risk - no protocol | High risk |
| Mohamed et al.^S137^ | 2021 | RCT | Low risk - Consented patients were equally randomized between the 2 parallel treatment arms using an online service (Sealed Envelope, London, UK). | Low risk - Consented patients were equally randomized between the 2 parallel treatment arms using an online service (Sealed Envelope, London, UK). | Low risk - not possible to blind participants and personelle to treatment group but unlikely to influence performance of the trial | Low risk - detailed duplex protocol and objective validated tools when clinical interpretation of results was required | Low risk - low attrition and balanced between groups | Low risk - registered protocol no deviations | Low risk |
| Rass et al.^S171^ | 2012 | RCT | Low risk - a computer-generated randomization list | Unclear - not mentioned whether concealed | Unclear - not stated | Unclear - outcome assessor was the surgeon performing the procedure | Unclear - attrition in each group not specified and patients treated per protocol | High risk - DVT not included as outcome in protocol | High risk |
| Onwudike et al.^S155^ | 2020 | RCT | Low risk - table of random numbers | Low risk - sealed opaque envelopes | High - study looked at compression vs no compression after RCT, there were no placebo-type stockings for the control group | Unclear - not stated | Low risk - low attrition in each group | Low risk - protocol registered | High risk |
| Rasmussen et al.^S169^ | 2011 | RCT | Low risk - randomisation in blocks of 12 | Low risk - sealed envelopes | Low risk - not possible to blind participants and personelle to treatment group but unlikely to influence performance of the trial | Unclear - outcome assessor was the surgeon performing the procedure | High risk - nearly 20% attrition in all groups with some at nearly 30% | High risk - no protocol | High risk |
| Doganci et al.^S58^ | 2010 | RCT | Unclear - randomised but not diuscussed how | Unclear - allocation concealment not specified | Unclear - not stated | Unclear - not stated | Low risk - no attrition | High risk - no protocol | High risk |
| Hirokawa et al.^S84^ | 2015 | RCT | Unclear - Each treatment was assigned to the patients according to the randomization list | Unclear risk - "The investigator in charge of study equipment assignment randomly assigned them in the order of enrollment at the respective institutions." | Low risk - not possible to blind participants and personelle to treatment group but unlikely to influence performance of the trial | Low risk - assessors not blinded but outcome of DVT unlikely to be influenced by knowledge of the intervention as measured with objective means | Low risk - no attrition | Low risk - protocol registered | Unclear risk |
| Mendes-Pinto et al.^S132^ | 2015 | RCT | High risk - "Patients were included in the study after a consultation by the vascular surgery team (PB, DMP, and LCBL)" | Unclear - allocation concealment not specified | Unclear - not stated | Unclear - not stated | Low risk - no atrittion | High risk - no protocol | High risk |
| Fischer et al.^S66^ | 2021 | RCT | High risk - the consecutively ordered patients were assigned to groups A, B or C in an alternating fashion until 50 patients were included in each group | High risk - allocation not concealed | Low risk - not possible to blind participants and personelle to treatment group but unlikely to influence performance of the trial | Low risk - assessors not blinded but outcome of DVT unlikely to be influenced by knowledge of the intervention as measured with objective means | Unclear risk - not stated how patients lost to follow up were analysed | High risk - no protocol | High risk |
| Gale et al.^S69^ | 2010 | RCT | Unclear - method of ranomisation not stated | Low risk - sealed envelope placed inpatient notes | Unclear - not stated | Unclear - not stated | Unclear risk - equal loss to follow up in each group but reasons not specified | High risk - no protocol | High risk |
| Christenson et al. ^S46^ | 2010 | RCT | Low risk - computerised randomisation software | Unclear - not stated | Low risk - independent surgeon | Low risk - independent angiologist | Low risk - low attrition | High risk - no protocol | High risk |
| Rasmussen et al.^S170^ | 2007 | RCT | Unclear risk - . The patients were all referred for varicose vein treatment by their family physician and were randomized in blocks of 10 sealed envelope | Low risk - randomized in blocks of 10 sealed envelope | Low risk - not possible to blind participants and personelle to treatment group but unlikely to influence performance of the trial | Unclear risk - the surgeon performing the procedure also performed the duplex | High risk - 27% of HL/S and 22% of EVL patients did not show up for 6 month follow up | High risk - no protocol | High risk |
| Mohammadi-Tofigh et al.^S138^ | 2020 | RCT | Low risk - simple randomization using the Rand function of Excel software (random number table). A mixture of numbers from 1 to 1090 was made, and patients entering the study got the next code from the table in order | Unclear risk - patients not aware of randomisation but not stated if clinicians were | Low risk - The evaluating team and the radiologist were all unaware of the patients’ group and were blinded. | Low risk - The evaluating team and the radiologist were all unaware of the patients’ group and were blinded. | Unclear risk - number followed up not specified | Low risk - registered protocol no deviations | Unclear risk |
| Samuel et al.^S177^ | 2013 | RCT | Low risk - randomized by sealed, opaque envelopes | Low risk - randomized by sealed, opaque envelopes | Unclear - not stated | Low risk - DUS fellow blinded | Low risk - equal attrition and interntion to treat | High risk - no protocol | High risk |
| Sydnor et al.^S194^ | 2016 | RCT | Unclear - "Randomization was performed in blocks of two, four, or six patients in order prevent ascertainment of allocations as well as to assure identical sizes of overall treatment groups" | Unclear - "Randomization was performed in blocks of two, four, or six patients in order prevent ascertainment of allocations as well as to assure identical sizes of overall treatment groups" | Low risk - Patients were blinded with regard to undergoing either EVLA or RFA. | Low risk - Objective data were interpreted and recorded by a nurse practitioner blinded with regard to the specific EVTA procedure (EVLA or RFA) performed on each patient. | Low risk - low attrition in each group over first 6 months when DVT outcome of interest | Unclear risk - reference protocol but no link nor evidence of it | Unclear risk |
| Nordon et al.^S149^ | 2011 | RCT | Low risk - binary random number tables | Low risk - sealed envelopes | Low risk - patients blinded to treatment received | Low risk - Patients were followed up at 1 week and 3 months after surgery by a physician blinded to treatment modality. | Low risk - no attrition | Low risk - registered protocol no deviations | Low risk |
| Watanabe et al.^S213^ | 2021 | RCT | Unclear risk - no information other than patients were randomised | Unclear risk - no information other than patients were randomised | Unclear risk - not stated | Low risk - aperformed by physician unaware of intervention | High risk - no intention to treat, no information on reasons for attrition and differential loss to follow up in each group | High risk - no protocol | High risk |
| Biemans et al.^S14^ | 2013 | RCT | Low risk - computerised risk by independent research nurse | Unclear risk - not stated whether allocation was concealed | Low risk - not possible to blind participants and personelle to treatment group but unlikely to influence performance of the trial | Low risk - ultrasound investigations were done by physicians not necessarily part of the research team | Low risk - intention to treat | Unclear risk - registered protocol but not all outcomes reported | Unclear risk |
| Almeida et al.^S3^ | 2009 | RCT | Low risk - Randomization was performed within 24 hours before the procedure and was accomplished by the investigators accessing a Web site and downloading the procedure to be performed. SAS software | High risk - Randomization was performed within 24 hours before the procedure and was accomplished by the investigators accessing a Web site and downloading the procedure to be performed. SAS software | Low risk - the actual treatment procedure was not discussed with the participants. | Unclear - not stated | Unclear risk - not stated how patients lost to follow up | High risk - no protocol | High risk |
| Beteli et al.^S13^ | 2018 | RCT | Low risk - odd numbers RFA even numbers EVLA | Low risk - sealed envelopes opened at time of induction of anaesthesia | Low risk - Patients and the evaluating physician were blinded to the technique employed. | Low risk - Patients and the evaluating physician were blinded to the technique employed. | Low risk - no attrition | Unclear risk - The study protocol and informed consent were approved by the local and national ethics committees but not accessible to reader | Unclear risk |
| Carradice et al.^S32^ | 2011 | RCT | Unclear risk - sealed opaque envelope from research nurse | Low risk - sealed opaque envelope from research nurse | Low risk - not possible to blind participants and personelle to treatment group but unlikely to influence performance of the trial | Low risk - assessors not blinded but outcome of DVT unlikely to be influenced by knowledge of the intervention as measured with objective means | Low risk - intention to treat | Low risk - registered protocol | Unclear risk |
| Disselhoff et al.^S57^ | 2008 | RCT | Low risk - numbered and sealed envelopes containing information about the type of treatment | Low risk - numbered and sealed envelopes containing information about the type of treatment | Low risk - not possible to blind participants and personelle to treatment group but unlikely to influence performance of the trial | Low risk - assessors not blinded but outcome of DVT unlikely to be influenced by knowledge of the intervention as measured with objective means | Low risk - low attrition, intention to treat used | Low risk - protocol registered | Unclear risk |
| Lugli et al.^S124^ | 2009 | RCT | [Low risk - allocated to two separate groups using a telephone randomization service.](https://journals.sagepub.com/doi/full/10.1258/phleb.2008.008045) | Low risk - the operating physician did not know to which group the patient was in until after the ELA had been performed | Low risk - not possible to blind participants and personelle to treatment group but unlikely to influence performance of the trial | Low risk - assessors not blinded but outcome of DVT unlikely to be influenced by knowledge of the intervention as measured with objective means | Low risk - low attrition | High risk - no protocol | High risk |
| Vuylsteke et al.^S211^ | 2010 | RCT | High risk - Randomization was done by alternately treating the patients with a 980 and a 1500 nm laser. | High risk - Randomization was done by alternately treating the patients with a 980 and a 1500 nm laser. | Low risk - not possible to blind participants and personelle to treatment group but unlikely to influence performance of the trial | Low risk - independent radiologist | Low risk - low attrition | High risk - no protocol | High risk |
| Flessenkamper et al.^S67^ | 2013 | RCT | Low risk - When patients were identified as fitting to the study and agreed to take part in it, they were randomly allocated to one of three groups by central telephone randomization in a 1:1:1 ratio. | [Low risk - A total of 500 lottery tickets were available in a lottery box in the central office of the study in Berlin managed by a secretary who had exclusive access rights](https://journals.sagepub.com/doi/10.1258/phleb.2011.011147) | Low risk - not possible to blind participants and personelle to treatment group but unlikely to influence performance of the trial | Unclear - not stated | Low risk - no attrition | Unclear risk - reference protocol but no link nor evidence of it | Unclear risk |
| Mese et al.^S135^ | 2015 | RCT | [Unclear - randomly assigned but not specified how](https://www.sciencedirect.com/science/article/abs/pii/S089050961500504X) | Unclear - allocation concealment not specified | Unclear - not stated if any blinding | Unclear - not stated | Low risk - no attrition | High risk - no protocol | High risk |
| Morrison et al.^S139^ | 2015 | RCT | [Low risk - Randomization was stratified by study site and used random block sizes of 4 or 6; assignments were obtained with an interactive voice response system linked to a web-based database.](https://www.sciencedirect.com/science/article/pii/S0741521414021855) | Low risk - Randomization was stratified by study site and used random block sizes of 4 or 6; assignments were obtained with an interactive voice response system linked to a web-based database. | Low risk - To reduce bias between groups, post-treatment stockings were worn by both CAE and RFA subjects because the RFA instructions for use require compression | Low risk - The assessment of vein closure could not be blinded to treatment because the ultrasonographic appearance of the implanted cyanoacrylate is unique and different from that observed after RFA treatment. Not expected to affect outcome | Low risk - low attrition in each group and intention to treat | High risk - protocol but not mentioned DVT as an outcome | High risk |
| ElKaffas et al.^S60^ | 2011 | RCT | Low risk - the included patients were asked to blindly choose an assignment card that would place them in either group A (RFA) or group B (surgical management). | Low risk - the included patients were asked to blindly choose an assignment card that would place them in either group A (RFA) or group B (surgical management). | Low risk - not possible to blind participants and personelle to treatment group but unlikely to influence performance of the trial | Low risk - not blinded but unclear if impact on outcome | Low risk - low attrition in each group up to 6 months | High risk - no protocol | High risk |
| Vahaaho et al.^S207^ | 2019 | RCT | Low risk - block randomization with sealed envelopes, into EVLA, RFA or MOCA in ratio of 1 : 1 : 2 | Low risk - block randomization with sealed envelopes, into EVLA, RFA or MOCA in ratio of 1 : 1 : 3 | Low risk - not possible to blind participants and personelle to treatment group but unlikely to influence performance of the trial | Unclear - not stated if outcome assesors blinded | Low risk - All patients attended the 1-month follow-up. At 1 year, 117 of the 125 remaining patients (93·6 per cent) attended the follow-up | Low risk - protocol registered | Unclear risk |
| Bootun et al.^S20^ | 2014 | RCT | Low risk - randomised to either MOCA (group A) or RFA (group B) truncal ablation using an online computerised service (Sealed Envelope, London, UK) | Low risk - randomised to either MOCA (group A) or RFA (group B) truncal ablation using an online computerised service (Sealed Envelope, London, UK) | Low risk - not possible to blind participants and personelle to treatment group but unlikely to influence performance of the trial | Low risk - No procedural blinding was possible due to the difference in techniques; however, the investigator at follow-up was blinded as to treatment group | High risk - very high attrition rate and no intention to treat nor explanation | Low risk - registered protocol | Unclear risk |
| Blaise et al.^S18^ | 2010 | RCT | Low risk - The randomisation was centralised and coordinated by a computer system. | Low risk - The randomisation was centralised and coordinated by a computer system. | Low risk - Envelopes were distributed to the investigating centres with numbers corresponding to the sclerosant lots. Double blinding was thus maintained for all the injections and until the end of patient follow-up | Low risk - The investigators performed both sclerotherapy and vascular sonography. The investigator and patient remained blinded to the treatment that was administered throughout the 3-year study period. | Low risk - low attrition in each gropup to 3 month follow up | Low risk - registered protocol | Low risk |
| Shadid et al. ^S181^ | 2012 | RCT | Low risk - computer-generated randomization scheme with random permuted blocks of eight | Unclear - not specified whether group allocation was concealed | Low risk - not possible to blind participants and personelle to treatment group but unlikely to influence performance of the trial | Low risk - outcomes were assessed objectively using colour duplex imaging by an analyst not involved in the study, but not stated whether they knew the trial arm | High risk - differential loss to follow up between groups without adequate explanation | High risk - protocol but not mentioned DVT as an outcome of interest | High risk |
| Tawfik et al.^S198^ | 2020 | RCT | Low risk - 100 eligible patients were allocated to one of the treatment arms using equal computerized randomization and sealed envelope technique | Low risk - 100 eligible patients were allocated to one of the treatment arms using equal computerized randomization and sealed envelope technique | Low risk - not possible to blind participants and personelle to treatment group but unlikely to influence performance of the trial | Low risk - Although blinding of the patients and clinical team was not possible owing to the nature of interventions, we managed to minimize probable bias by recruiting preoperative and postoperative assessors who were not aware of the available treatment options | Low risk - no attrition over 12 months | Unclear - reference to study protocol but no registration number provided | Unclear risk |
| Lattimer et al.^S116^ | 2012 | RCT | Low risk - Randomisation was performed with numbered sealed envelopes. | Low risk - Randomisation was performed with numbered sealed envelopes. | Unclear - not stated if any blinding | Unclear - not stated if any blinding of outcome assessment | Unclear - equal attrition across both groups but reasons for attrition not stated | Low risk - protocol registered | Unclear risk |
| Bozoglan et al.^S24^ | 2016 | RCT | Low risk - Patients were randomized for EVLA and RFA. The EVLA was first performed on 1 patient and RFA first on the next. No patient was aware which procedure would be performed on which leg. | Unclear risk - no information other than patients were randomised | Low risk - No patient was aware which procedure would be performed on which leg. | Unclear - not stated if any blinding of outcome assessment | Low risk - no attrition | High risk - no protocol | High risk |

**References for Supplemental Digital Content**

1. Abbassi-Ghadi N, Hafez H. Ultrasound-guided foam sclerotherapy within a rolling treatment programme is an effective low-cost treatment for superficial venous insufficiency. *Phlebology*. Jun 2013;28(4):195-200.
2. Agus GB, Mancini S, Magi G, et al. The first 1000 cases of Italian Endovenous-laser Working Group (IEWG). Rationale, and long-term outcomes for the 1999-2003 period. *International Angiology* 2006; 25(2):209-215.
3. Almeida JI, Kaufman J, Gockeritz O, et al. Radiofrequency endovenous ClosureFAST versus laser ablation for the treatment of great saphenous reflux: a multicenter, single-blinded, randomized study (RECOVERY study). *J Vasc Interv Radiol* 2009; 20(6):752-759.
4. Arslan U, Calik E, Tort M, et al. More Successful Results with Less Energy in Endovenous Laser Ablation Treatment: Long-term Comparison of Bare-tip Fiber 980 nm Laser and Radial-tip Fiber 1470 nm Laser Application. *Ann Vasc Surg* 2017; 45:166-172.
5. Altin FH, Aydin S, Erkoc K, et al. Endovenous laser ablation for saphenous vein insufficiency: short- and mid-term results of 230 procedures. *Vascular* 2015; 23(1):3-8.
6. Aurshina A, Zhang Y, Wang F, et al. The effect of increasing catheter distance from the deep junction on the outcomes of radiofrequency vein ablation. *Journal of Vascular Surgery* 2018; 6(5):614-620.
7. Baccellieri D, Apruzzi L, Ardita V, et al. Early results of mechanochemical ablation for small saphenous vein incompetency using 2% polidocanol. *Journal of Vascular Surgery* 2021; 9(3):683-690.
8. Bademci MS, Tayfur K, Ocakoglu G, et al. A new percutaneous technique: N-butyl cyanoacrylate adhesive for the treatment of giant saphenous vein insufficiency. *Vascular* 2018; 26(2):194-197.
9. Barker T, Evison F, Benson R, et al. Risk of venous thromboembolism following surgical treatment of superficial venous incompetence. *Vasa* 2017; 46(6):484-489.
10. Belcaro G, Nicolaides AN, Ricci A, et al. Endovascular sclerotherapy, surgery, and surgery plus sclerotherapy in superficial venous incompetence: a randomized, 10-year follow-up trial--final results. *Angiology* 2000; 51(7):529-534.
11. Benarroch-Gampel J, Sheffield KM, Boyd CA, et al. Analysis of venous thromboembolic events after saphenous ablation. *Journal of Vascular Surgery* 2013; 1(1):26-32.
12. Bergan J, Pascarella L, Mekenas L. Venous disorders: treatment with sclerosant foam. *Journal of Cardiovascular Surgery* 2006; 47(1):9-18.
13. Beteli CB, Rossi FH, de Almeida BL, et al. Prospective, double-blind, randomized controlled trial comparing electrocoagulation and radiofrequency in the treatment of patients with great saphenous vein insufficiency and lower limb varicose veins. *J Vasc Surg Venous Lymphat Disord* 2018; 6(2):212-219.
14. Biemans AA, Kockaert M, Akkersdijk GP, et al. Comparing endovenous laser ablation, foam sclerotherapy, and conventional surgery for great saphenous varicose veins. *J Vasc Surg* 2013; 58(3):727-734 e1.
15. Bisang U, Meier TO, Enzler M, et al. Results of endovenous ClosureFast treatment for varicose veins in an outpatient setting. *Phlebology* 2012; 27(3):118-123.
16. Bishawi M, Bernstein R, Boter M, et al. Mechanochemical ablation in patients with chronic venous disease: a prospective multicenter report. *Phlebology* 2014; 29(6):397-400.
17. Bitargil M, El Kilic H. Our experience regarding patients with headache, vomiting, and urinary retention following endothermal ablation of the greater saphenous vein under spinal anesthesia: Gender type, age interval, and procedural risk factors are important. *Vascular* 2020; 28(5):591-596.
18. Blaise S, Bosson JL, Diamand JM. Ultrasound-guided sclerotherapy of the great saphenous vein with 1% vs. 3% polidocanol foam: a multicentre double-blind randomised trial with 3-year follow-up. *Eur J Vasc Endovasc Surg* 2010; 39(6):779-786.
19. Boersma D, van Eekeren RR, Werson DA, et al. Mechanochemical endovenous ablation of small saphenous vein insufficiency using the ClariVein((R)) device: one-year results of a prospective series. *Eur J Vasc Endovasc Surg* 2013; 45(3):299-303.
20. Bootun R, Lane TR, Dharmarajah B, et al. Intra-procedural pain score in a randomised controlled trial comparing mechanochemical ablation to radiofrequency ablation: The Multicentre Venefit versus ClariVein(R) for varicose veins trial. *Phlebology* 2016; 31(1):61-65.
21. Borghese O, Pisani A, Di Centa I. Endovenous radiofrequency for chronic superficial venous insufficiency: Clinical outcomes and impact in quality of life. *Journal De Medecine Vasculaire* 2021; 46(1):3-8.
22. Boros MJ, O'Brien SP, McLaren JT, et al. High ligation of the saphenofemoral junction in endovenous obliteration of varicose veins. *Vascular & Endovascular Surgery* 2008; 42(3):235-238.
23. Bozkurt AK, Yilmaz MF. A prospective comparison of a new cyanoacrylate glue and laser ablation for the treatment of venous insufficiency. *Phlebology* 2016; 31(1 Suppl):106-113.
24. Bozoglan O, Mese B, Eroglu E, et al. Comparison of Endovenous Laser and Radiofrequency Ablation in Treating Varicose Veins in the Same Patient. *Vasc Endovascular Surg* 2016; 50(1):47-51.
25. Bradbury AW, Bate G, Pang K, et al. Ultrasound-guided foam sclerotherapy is a safe and clinically effective treatment for superficial venous reflux. *J Vasc Surg* 2010; 52(4):939-945.
26. Brown CS, Obi AT, Cronenwett JL, et al. Outcomes after truncal ablation with or without concomitant phlebectomy for isolated symptomatic varicose veins (C2 disease). *J Vasc Surg Venous Lymphat Disord* 2021; 9(2):369-376.
27. Cabrera J, Redondo P, Becerra A, et al. Ultrasound-guided injection of polidocanol microfoam in the management of venous leg ulcers. *Arch Dermatol* 2004; 140(6):667-673.
28. Cabrero Fernandez M, Martinez Lopez I, Hernandez Mateo MM, et al. Prospective study of safety and effectiveness in the use of radiofrequency ablation for incompetent great saphenous vein >/=12 mm. *J Vasc Surg Venous Lymphat Disord* 2017; 5(6):810-816.
29. Calcagno D, Rossi JA, Ha C. Effect of saphenous vein diameter on closure rate with ClosureFAST radiofrequency catheter. *Vasc Endovascular Surg* 2009; 43(6):567-570.
30. Campos Gomes CV, Prado Nunes MA, Navarro TP, et al. Elastic compression after ultrasound-guided foam sclerotherapy in overweight patients does not improve primary venous hemodynamics outcomes. *Journal of Vascular Surgery* 2020; 8(1):110-117.
31. Can Caliskan K, Cakmakci E, Celebi I, et al. Endovenous 1470 nm laser treatment of the saphenous vein: early report of pain assessment. *Journal of Cardiovascular Surgery* 2013; 54(2):263-267.
32. Carradice D, Mekako AI, Mazari FA, et al. Randomized clinical trial of endovenous laser ablation compared with conventional surgery for great saphenous varicose veins. *Br J Surg* 2011; 98(4):501-510.
33. Carruthers TN, Farber A, Rybin D, et al. Interventions on the superficial venous system for chronic venous insufficiency by surgeons in the modern era: an analysis of ACS-NSQIP. *Vascular & Endovascular Surgery* 2014; 48(7-8):482-490.
34. Casana R, Tolva VS, Odero A, Jr., et al. Three-year follow-up and quality of life of endovenous radiofrequency ablation of the great saphenous vein with the ClosureFast TM procedure: Influence of BMI and CEAP class. *Vascular* 2018; 26(5):498-508.
35. Cavezzi AF, A.; Ricci, S.; Tessari, L. Treatment of Varicose Veins by Foam Sclerotherapy: Two Clinical Series. *Phlebology* 2002; 17(1):13-18.
36. Cavezzi A, Mosti G, Campana F, et al. Catheter Foam Sclerotherapy of the Great Saphenous Vein, with Perisaphenous Tumescence Infiltration and Saphenous Irrigation. *European Journal of Vascular & Endovascular Surgery* 2017; 54(5):629-635.
37. Chaar CI, Hirsch SA, Cwenar MT, et al. Expanding the role of endovenous laser therapy: results in large diameter saphenous, small saphenous, and anterior accessory veins. *Annals of Vascular Surgery* 2011; 25(5):656-661.
38. Chan SSJ, Yap CJQ, Tan SG, et al. The utility of endovenous cyanoacrylate glue ablation for incompetent saphenous veins in the setting of venous leg ulcers. *Journal of Vascular Surgery* 2020; 8(6):1041-1048.
39. Chan YC, Law Y, Cheung GC, et al. Predictors of Recanalization for Incompetent Great Saphenous Veins Treated with Cyanoacrylate Glue. *J Vasc Interv Radiol* 2017; 28(5):665-671.
40. Chandler JGP, O; Sessa, C; Schuller-Petrovicc, S; Kabnick, S; Bergan, J. Treatment of Primary Venous Insufficiency by Endovenous Saphenous Vein Obliteration. *Vascular and Endovascular Surgery* 2000; 34(3):201-214.
41. Chang SL, Hu S, Huang YL, et al. Treatment of Varicose Veins Affects the Incidences of Venous Thromboembolism and Peripheral Artery Disease. *Circulation: Cardiovascular Interventions* 2021; 14(3):e010207.
42. Chen JQ, Xie H, Deng HY, et al. Endovenous laser ablation of great saphenous vein with ultrasound-guided perivenous tumescence: early and midterm results. *Chinese Medical Journal* 2013; 126(3):421-425.
43. Chi YW, Woods TC. Clinical risk factors to predict deep venous thrombosis post-endovenous laser ablation of saphenous veins. *Phlebology* 2014; 29(3):150-153.
44. Cho S, Gibson K, Lee SH, et al. Incidence, classification, and risk factors of endovenous glue-induced thrombosis after cyanoacrylate closure of the incompetent saphenous vein. *Journal of Vascular Surgery* 2020; 8(6):991-998.
45. Choi JH, Park HC, Joh JH. The occlusion rate and patterns of saphenous vein after radiofrequency ablation. *J Korean Surg Soc* 2013; 84(2):107-113.
46. Christenson JT, Gueddi S, Gemayel G, et al. Prospective randomized trial comparing endovenous laser ablation and surgery for treatment of primary great saphenous varicose veins with a 2-year follow-up. *J Vasc Surg* 2010; 52(5):1234-1241.
47. Ciostek PK, M.; Wozniak, W.; Mitek, T.; Myrcha, P.; Migda, B. . Phlebogriffe – a new device for mechanochemical ablation of incompetent saphenous veins: a pilot study. *Phlebological Review* 2015; 23(3):72-77.
48. Creton D, Pichot O, Sessa C, et al. Radiofrequency-powered segmental thermal obliteration carried out with the ClosureFast procedure: results at 1 year. *Ann Vasc Surg* 2010; 24(3):360-366.
49. Cuffolo G, Hardy E, Perkins J, et al. The effects of foam sclerotherapy on ulcer healing: a single-centre prospective study. *Annals of the Royal College of Surgeons of England* 2019; 101(4):285-289.
50. Darvall KA, Bate GR, Silverman SH, et al. Medium-term results of ultrasound-guided foam sclerotherapy for small saphenous varicose veins. *British Journal of Surgery* 2009; 96(11):1268-1273.
51. de Aguiar ET, Dos Santos JB, Carvalho DD. Venous thromboembolism after ultrasound guided foam sclerotherapy. *Phlebology* 2021; 36(3):233-239.
52. de Araujo WJB, Timi JRR, Nejm CSJ, et al. Persistent below-knee great saphenous vein reflux after above-knee endovenous laser ablation with 1470-nm laser: a prospective study. *J Vasc Bras* 2016; 15(2):113-119.
53. de Oliveira RG, de Morais Filho D, Engelhorn CA, et al. Foam sclerotherapy for lower-limb varicose veins: impact on saphenous vein diameter. *Radiologia Brasileira* 2018; 51(6):372-376.
54. Desmyttere J, Grard C, Wassmer B, et al. Endovenous 980-nm laser treatment of saphenous veins in a series of 500 patients. *Journal of Vascular Surgery* 2007; 46(6):1242-1247.
55. Desmyttere J, Grard C, Stalnikiewicz G, et al. Endovenous laser ablation (980 nm) of the small saphenous vein in a series of 147 limbs with a 3-year follow-up. *European Journal of Vascular & Endovascular Surgery* 2010; 39(1):99-103.
56. Disselhoff BCVM, Der Kinderen DJ, Moll FL. Is there recanalization of the great saphenous vein 2 years after endovenous laser treatment? *Journal of Endovascular Therapy* 2005; 12(6):731-738.
57. Disselhoff BC, der Kinderen DJ, Kelder JC, et al. Randomized clinical trial comparing endovenous laser with cryostripping for great saphenous varicose veins. *Br J Surg* 2008; 95(10):1232-1238.
58. Doganci S, Demirkilic U. Comparison of 980 nm laser and bare-tip fibre with 1470 nm laser and radial fibre in the treatment of great saphenous vein varicosities: a prospective randomised clinical trial. *Eur J Vasc Endovasc Surg* 2010; 40(2):254-259.
59. Dunn CW, Kabnick LS, Merchant RF, et al. Endovascular radiofrequency obliteration using 90 degrees C for treatment of great saphenous vein. *Annals of Vascular Surgery* 2006; 20(5):625-629.
60. Helmy ElKaffas K, ElKashef O, ElBaz W. Great saphenous vein radiofrequency ablation versus standard stripping in the management of primary varicose veins-a randomized clinical trial. *Angiology* 2011; 62(1):49-54.
61. Ergenoglu MU, Sayin M, Kucukaksu S. Fate of vena saphena magna stump after endovenous laser ablation with 980-nm diode laser: 12-month follow-up. *Photomedicine and Laser Surgery* 2010; 28(5):659-662.
62. Ergenoglu MU, Sayin MM, Kucukaksu DS. Endovenous laser ablation with 980-nm diode laser: early and midterm results. *Photomedicine and Laser Surgery* 2011; 29(10):691-697.
63. Eroglu E, Yasim A. A Randomised Clinical Trial Comparing N-Butyl Cyanoacrylate, Radiofrequency Ablation and Endovenous Laser Ablation for the Treatment of Superficial Venous Incompetence: Two Year Follow up Results. *European Journal of Vascular & Endovascular Surgery* 2018; 56(4):553-560.
64. Fernandez CF, Roizental M, Carvallo J. Combined endovenous laser therapy and microphlebectomy in the treatment of varicose veins: Efficacy and complications of a large single-center experience. *Journal of Vascular Surgery* 2008; 48(4):947-952.
65. Fernando RS, Muthu C. Adoption of endovenous laser treatment as the primary treatment modality for varicose veins: the Auckland City Hospital experience. *New Zealand Medical Journal* 2014; 127(1399):43-50.
66. Fischer L, Maurins U, Rabe E, et al. Effect of Compression Stockings after Endovenous Laser Ablation of the Great Saphenous Vein with a 1470 nm Diode Laser Device and a 2ring Fiber. *J Clin Med* 2021; 10(17).
67. Flessenkamper I, Hartmann M, Stenger D, et al. Endovenous laser ablation with and without high ligation compared with high ligation and stripping in the treatment of great saphenous varicose veins: initial results of a multicentre randomized controlled trial. *Phlebology* 2013; 28(1):16-23.
68. Gabriel V, Jimenez JC, Alktaifi A, et al. Success of endovenous saphenous and perforator ablation in patients with symptomatic venous insufficiency receiving long-term warfarin therapy. *Ann Vasc Surg* 2012; 26(5):607-611.
69. Gale SS, Lee JN, Walsh ME, et al. A randomized, controlled trial of endovenous thermal ablation using the 810-nm wavelength laser and the ClosurePLUS radiofrequency ablation methods for superficial venous insufficiency of the great saphenous vein. *J Vasc Surg* 2010; 52(3):645-650.
70. Garcia-Madrid C, Pastor Manrique JO, Sanchez VA, et al. Endovenous radiofrequency ablation (venefit procedure): impact of different energy rates on great saphenous vein shrinkage. *Annals of Vascular Surgery* 2013; 27(3):314-321.
71. Gibson K, Kabnick L, Varithena 013 Investigator G. A multicenter, randomized, placebo-controlled study to evaluate the efficacy and safety of Varithena(R) (polidocanol endovenous microfoam 1%) for symptomatic, visible varicose veins with saphenofemoral junction incompetence. *Phlebology* 2017; 32(3):185-193.
72. Gibson KD, Ferris BL, Polissar N, et al. Endovenous laser treatment of the small [corrected] saphenous vein: efficacy and complications. *J Vasc Surg* 2007; 45(4):795-801; discussion 801-3.
73. Gifford SM, Kalra M, Gloviczki P, et al. Reflux in the below-knee great saphenous vein can be safely treated with endovenous ablation. *J Vasc Surg Venous Lymphat Disord* 2014; 2(4):397-402.
74. Gillet JL, Desnos CH, Lausecker M, et al. Sclerotherapy is a safe method of treatment of chronic venous disorders in older patients: A prospective and comparative study of consecutive patients. *Phlebology* 2017; 32(4):234-240.
75. Gillet JL, Guedes JM, Guex JJ, et al. Side-effects and complications of foam sclerotherapy of the great and small saphenous veins: a controlled multicentre prospective study including 1,025 patients. *Phlebology* 2009; 24(3):131-138.
76. Gillet JL, Lausecker M, Sica M, et al. Is the treatment of the small saphenous veins with foam sclerotherapy at risk of deep vein thrombosis? *Phlebology* 2014; 29(9):600-607.
77. Gohel MS, Heatley F, Liu X, et al. Early versus deferred endovenous ablation of superficial venous reflux in patients with venous ulceration: the EVRA RCT. *Health Technology Assessment (Winchester, England)* 2019; 23(24):1-96.
78. Golbasi I, Turkay C, Erbasan O, et al. Endovenous laser with miniphlebectomy for treatment of varicose veins and effect of different levels of laser energy on recanalization. A single center experience. *Lasers in Medical Science* 2015; 30(1):103-108.
79. Hamel-Desnos C, Gerard JL, Desnos P. Endovenous laser procedure in a clinic room: feasibility and side effects study of 1,700 cases. *Phlebology* 2009; 24(3):125-130.
80. Hamel-Desnos CM, Gillet JL, Desnos PR, et al. Sclerotherapy of varicose veins in patients with documented thrombophilia: a prospective controlled randomized study of 105 cases. *Phlebology* 2009; 24(4):176-182.
81. Haqqani OP, Vasiliu C, O'Donnell TF, et al. Great saphenous vein patency and endovenous heat-induced thrombosis after endovenous thermal ablation with modified catheter tip positioning. *J Vasc Surg* 2011; 54(6 Suppl):10S-17S.
82. Harlander-Locke M, Jimenez JC, Lawrence PF, et al. Management of endovenous heat-induced thrombus using a classification system and treatment algorithm following segmental thermal ablation of the small saphenous vein. *Journal of Vascular Surgery* 2013; 58(2):427-431.
83. Hingorani AP, Ascher E, Markevich N, et al. Deep venous thrombosis after radiofrequency ablation of greater saphenous vein: a word of caution. *Journal of Vascular Surgery* 2004; 40(3):500-504.
84. Hirokawa M, Ogawa T, Sugawara H, et al. Comparison of 1470 nm Laser and Radial 2ring Fiber with 980 nm Laser and Bare-Tip Fiber in Endovenous Laser Ablation of Saphenous Varicose Veins: A Multicenter, Prospective, Randomized, Non-Blind Study. *Ann Vasc Dis* 2015; 8(4):282-289.
85. Holewijn S, van Eekeren R, Vahl A, et al. Two-year results of a multicenter randomized controlled trial comparing Mechanochemical endovenous Ablation to RADiOfrequeNcy Ablation in the treatment of primary great saphenous vein incompetence (MARADONA trial). *Journal of Vascular Surgery* 2019; 7(3):364-374.
86. Huisman LC, Bruins RM, van den Berg M, et al. Endovenous laser ablation of the small saphenous vein: prospective analysis of 150 patients, a cohort study. *Eur J Vasc Endovasc Surg* 2009; 38(2):199-202.
87. Itoga NK, Rothenberg KA, Deslarzes-Dubuis C, et al. Incidence and Risk Factors for Deep Vein Thrombosis after Radiofrequency and Laser Ablation of the Lower Extremity Veins. *Ann Vasc Surg* 2020; 62:45-50 e2.
88. Izumi M, Ikeda Y, Yamashita H, et al. Safety and Effectiveness of Endovenous Laser Ablation Combined With Ligation for Severe Saphenous Varicose Veins in Japanese Patients. *International Heart Journal* 2016; 57(1):87-90.
89. Jacobs CE, Pinzon MM, Orozco J, et al. Deep venous thrombosis after saphenous endovenous radiofrequency ablation: is it predictable? *Annals of Vascular Surgery* 2014; 28(3):679-685.
90. Janne d'Othee B, Walker TG, Kalva SP, et al. Endovenous laser ablation of the small saphenous vein sparing the saphenopopliteal junction. *Cardiovascular & Interventional Radiology* 2010; 33(4):766-771.
91. Jimenez JC, Lawrence PF, Pavlyha M, et al. Endovenous microfoam ablation of below knee superficial truncal veins is safe and effective in patients with prior saphenous treatment across a wide range of CEAP classes. *J Vasc Surg Venous Lymphat Disord* 2022; 10(2):390-394.
92. Jimenez JC, Lawrence PF, Woo K, et al. Adjunctive techniques to minimize thrombotic complications following microfoam sclerotherapy of saphenous trunks and tributaries. *Journal of Vascular Surgery* 2021; 9(4):904-909.
93. Jung IM, Min SI, Heo SC, et al. Combined endovenous laser treatment and ambulatory phlebectomy for the treatment of saphenous vein incompetence. *Phlebology* 2008; 23(4):172-177.
94. Kane K, Fisher T, Bennett M, et al. The incidence and outcome of endothermal heat-induced thrombosis after endovenous laser ablation. *Annals of Vascular Surgery* 2014; 28(7):1744-1750.
95. Kapoor A, Kapoor A, Mahajan G. Endovenous ablation of saphenofemoral insufficiency: analysis of 100 patients using RF closure fast technique. *Indian J Surg* 2010; 72(6):458-462.
96. Karathanos C, Spanos K, Batzalexis K, et al. Prospective comparative study of different endovenous thermal ablation systems for treatment of great saphenous vein reflux. *J Vasc Surg Venous Lymphat Disord* 2021; 9(3):660-668.
97. Keo HH, Spinedi L, Staub D, et al. Safety and efficacy of outpatient endovenous laser ablation in patients 75 years and older: a propensity score-matched analysis. *Swiss Medical Weekly* 2019; 149:w20083.
98. Keo HH, Baumann F, Diehm N, et al. Rivaroxaban versus fondaparinux for thromboprophylaxis after endovenous laser ablation. *Journal of Vascular Surgery* 2017; 5(6):817-823.
99. Keo HH, Spinedi L, Staub D, et al. Duration of pharmacological thromboprophylaxis after outpatient endovenous laser ablation: a propensity score-matched analysis. *Swiss Medical Weekly* 2019; 149:w20166.
100. Khan Kharl RA, Khan NI, Pervaiz HK, et al. Foam Sclerotherapy: An Emerging, Minimally Invasive And Safe Modality Of Treatment For Varicose Veins. *Journal of Ayub Medical College, Abbottabad: JAMC* 2019; 31(Suppl 1)(4):S641-S645.
101. Kim PS, Elias S, Gasparis A, et al. Results of polidocanol endovenous microfoam in clinical practice. *J Vasc Surg Venous Lymphat Disord* 2021; 9(1):122-127.
102. Kim JS, Park SW, Yun IJ, et al. Retrograde Endovenous Laser Ablation through Saphenopopliteal Junctional Area for Incompetent Small Saphenous Vein: Comparison with Antegrade Approach. *Korean J Radiol* 2016; 17(3):364-369.
103. King JT, O'Byrne M, Vasquez M, et al. Treatment of Truncal Incompetence and Varicose Veins with a Single Administration of a New Polidocanol Endovenous Microfoam Preparation Improves Symptoms and Appearance. *Eur J Vasc Endovasc Surg* 2015; 50(6):784-793.
104. King T, Coulomb G, Goldman A, et al. Experience with concomitant ultrasound-guided foam sclerotherapy and endovenous laser treatment in chronic venous disorder and its influence on Health Related Quality of Life: interim analysis of more than 1000 consecutive procedures. *International Angiology* 2009; 28(4):289-297.
105. Knipp BS, Blackburn SA, Bloom JR, et al. Endovenous laser ablation: venous outcomes and thrombotic complications are independent of the presence of deep venous insufficiency. *Journal of Vascular Surgery* 2008; 48(6):1538-1545.
106. Kontothanassis D, Di Mitri R, Ferrari Ruffino S, et al. Endovenous laser treatment of the small saphenous vein. *J Vasc Surg* 2009; 49(4):973-979 e1.
107. Koramaz I, El Kilic H, Gokalp F, et al. Ablation of the great saphenous vein with nontumescent n-butyl cyanoacrylate versus endovenous laser therapy. *J Vasc Surg Venous Lymphat Disord* 2017; 5(2):210-215.
108. Korkmaz K, Yener AU, Gedik HS, et al. Tumescentless endovenous radiofrequency ablation with local hypothermia and compression technique. *Cardiovasc J Afr* 2013; 24(8):313-317.
109. Kulkarni SR, Messenger DE, Slim FJ, et al. The incidence and characterization of deep vein thrombosis following ultrasound-guided foam sclerotherapy in 1000 legs with superficial venous reflux. *J Vasc Surg Venous Lymphat Disord* 2013; 1(3):231-238.
110. Kulkarni SR, Slim FJ, Emerson LG, et al. Effect of foam sclerotherapy on healing and long-term recurrence in chronic venous leg ulcers. *Phlebology* 2013; 28(3):140-146.
111. Kurihara N, Hirokawa M, Yamamoto T. Postoperative Venous Thromboembolism in Patients Undergoing Endovenous Laser and Radiofrequency Ablation of the Saphenous Vein. *Avd* 2016; 9(4):259-266.
112. Kurnicki J, Oseka M, Tworus R, et al. Ultrasound-guided foam sclerotherapy of great saphenous vein with 2% polidocanol - one-year follow-up results. *Wideochirurgia i Inne Techniki Maloinwazyjne* 2016; 11(2):67-75.
113. Kutas B, Ozdemir F, Tezcan O, et al. Does the direction of tumescent solution delivery matter in endovenous laser ablation of the great saphenous vein? *Ther Adv Cardiovasc Dis* 2015; 9(6):397-402.
114. Lakhwani MN, Dadlani NI, Wong YC. 980-nm laser therapy versus varicose vein surgery in racially diverse Penang, Malaysia. *ANZ Journal of Surgery* 2009; 79(5):352-357.
115. Lane T, Bootun R, Dharmarajah B, et al. A multi-centre randomised controlled trial comparing radiofrequency and mechanical occlusion chemically assisted ablation of varicose veins - Final results of the Venefit versus Clarivein for varicose veins trial. *Phlebology* 2017; 32(2):89-98.
116. Lattimer CR, Azzam M, Kalodiki E, et al. Cost and effectiveness of laser with phlebectomies compared with foam sclerotherapy in superficial venous insufficiency. Early results of a randomised controlled trial. *Eur J Vasc Endovasc Surg* 2012; 43(5):594-600.
117. Lawrence PF, Chandra A, Wu M, et al. Classification of proximal endovenous closure levels and treatment algorithm. *J Vasc Surg* 2010; 52(2):388-393.
118. Lawson JA, Gauw SA, van Vlijmen CJ, et al. Prospective comparative cohort study evaluating incompetent great saphenous vein closure using radiofrequency-powered segmental ablation or 1470-nm endovenous laser ablation with radial-tip fibers (Varico 2 study). *J Vasc Surg Venous Lymphat Disord* 2018; 6(1):31-40.
119. Leopardi M, Salerno A, Dante A, et al. Endovenous Laser Ablation with 1,470-nm Diode with Tumescence Anesthesia and Saphenofemoral Ligation: Propensity Score Match Comparison. *Annals of Vascular Surgery* 2019; 58:302-308.
120. Li X, Yang B, Li X, et al. Prospective Comparison of Effect of Ligation and Foam Sclerotherapy with Foam Sclerotherapy Alone for Varicose Veins. *Annals of Vascular Surgery* 2018; 49:75-79.
121. Liang Y, Jia Y, Zhang Z, et al. A novel endovenous laser ablation strategy of treatment of greater saphenous vein varicosities with difficult wire placement. *International journal of clinical and experimental medicine* 2015; 8(4):5831-5838.
122. Lin JC, Peterson EL, Rivera ML, et al. Vein mapping prior to endovenous catheter ablation of the great saphenous vein predicts risk of endovenous heat-induced thrombosis. *Vasc Endovascular Surg* 2012; 46(5):378-383.
123. Lobastov K, Vorontsova A, Bargandzhiya A, et al. The frequency and clinical significance of nontarget superficial and deep vein occlusion after physician compounded foam sclerotherapy of varicose tributaries. *Phlebology* 2020; 35(6):430-439.
124. Lugli M, Cogo A, Guerzoni S, et al. Effects of eccentric compression by a crossed-tape technique after endovenous laser ablation of the great saphenous vein: a randomized study. *Phlebology* 2009; 24(4):151-156.
125. Lurie F, Kistner RL. Pretreatment elevated D-dimer levels without systemic inflammatory response are associated with thrombotic complications of thermal ablation of the great saphenous vein. *J Vasc Surg Venous Lymphat Disord* 2013; 1(2):154-158.
126. Lurie F, Creton D, Eklof B, et al. Prospective randomized study of endovenous radiofrequency obliteration (closure procedure) versus ligation and stripping in a selected patient population (EVOLVeS Study). *Journal of Vascular Surgery* 2003; 38(2):207-214.
127. Manfrini S, Gasbarro V, Danielsson G, et al. Endovenous management of saphenous vein reflux. Endovenous Reflux Management Study Group. *J Vasc Surg* 2000; 32(2):330-342.
128. Mao J, Zhang C, Wang Z, et al. A retrospective study comparing endovenous laser ablation and microwave ablation for great saphenous varicose veins. *Eur Rev Med Pharmacol Sci* 2012; 16(7):873-877.
129. Marsh P, Price BA, Holdstock J, et al. Deep vein thrombosis (DVT) after venous thermoablation techniques: rates of endovenous heat-induced thrombosis (EHIT) and classical DVT after radiofrequency and endovenous laser ablation in a single centre. *European Journal of Vascular & Endovascular Surgery* 2010; 40(4):521-527.
130. Memetoglu ME, Kurtcan S, Erbasan O, et al. Endovenous ablation with a 940 nm laser for the treatment of great saphenous vein insufficiency: short- to mid-term results. *Diagnostic & Interventional Radiology* 2012; 18(1):106-110.
131. Memetoglu ME, Yilmaz M, Kehlibar T, et al. Ultrasonography-guided foam sclerotherapy in patients with small saphenous vein insufficiency. *Journal of Vascular Surgery* 2020; 8(5):799-804.
132. Mendes-Pinto D, Bastianetto P, Cavalcanti Braga Lyra L, et al. Endovenous laser ablation of the great saphenous vein comparing 1920-nm and 1470-nm diode laser. *Int Angiol* 2016; 35(6):599-604.
133. Merchant RF, Pichot O, Closure Study G. Long-term outcomes of endovenous radiofrequency obliteration of saphenous reflux as a treatment for superficial venous insufficiency. *J Vasc Surg* 2005; 42(3):502-509; discussion 509.
134. Merchant RF, DePalma RG, Kabnick LS. Endovascular obliteration of saphenous reflux: a multicenter study. *J Vasc Surg* 2002; 35(6):1190-1196.
135. Mese B, Bozoglan O, Eroglu E, et al. A Comparison of 1,470-nm Endovenous Laser Ablation and Radiofrequency Ablation in the Treatment of Great Saphenous Veins 10 mm or More in Size. *Ann Vasc Surg* 2015; 29(7):1368-72.
136. Min RJ, Khilnani N, Zimmet SE. Endovenous laser treatment of saphenous vein reflux: long-term results. *Journal of Vascular & Interventional Radiology* 2003; 14(8):991-996.
137. Mohamed AH, Leung C, Wallace T, et al. A Randomized Controlled Trial of Endovenous Laser Ablation Versus Mechanochemical Ablation With ClariVein in the Management of Superficial Venous Incompetence (LAMA Trial). *Annals of Surgery* 2021; 273(6):e188-e195.
138. Mohammadi Tofigh A, Tahmasebi H, Zebarjadi J. Comparing the Success Rate and Side Effects of Endovenous Laser Ablation and Radiofrequency Ablation to Treat Varicose Veins in the Lower Limbs: A Randomized Clinical Trial. *J Lasers Med Sci* 2020; 11(Suppl 1):S43-S48.
139. Morrison N, Gibson K, McEnroe S, et al. Randomized trial comparing cyanoacrylate embolization and radiofrequency ablation for incompetent great saphenous veins (VeClose). *J Vasc Surg* 2015; 61(4):985-994.
140. Moul DK, Housman L, Romine S, et al. Endovenous laser ablation of the great and short saphenous veins with a 1320-nm neodymium:yttrium-aluminum-garnet laser: retrospective case series of 1171 procedures. *Journal of the American Academy of Dermatology* 2014; 70(2):326-331.
141. Mozes G, Kalra M, Carmo M, et al. Extension of saphenous thrombus into the femoral vein: a potential complication of new endovenous ablation techniques. *Journal of Vascular Surgery* 2005; 41(1):130-135.
142. Murli NL, Lee TC, Beh ML. Holistic management of venous ulcers especially with endovenous laser treatment using 980nm laser in an ethnically diverse society. *Medical Journal of Malaysia* 2013; 68(6):453-458.
143. Myers KA, Jolley D, Clough A, et al. Outcome of ultrasound-guided sclerotherapy for varicose veins: medium-term results assessed by ultrasound surveillance. *European Journal of Vascular & Endovascular Surgery* 2007; 33(1):116-121.
144. Myers KA, Jolley D. Factors affecting the risk of deep venous occlusion after ultrasound-guided sclerotherapy for varicose veins. *European Journal of Vascular & Endovascular Surgery* 2008; 36(5):602-605.
145. Nael R, Rathbun S. Effectiveness of foam sclerotherapy for the treatment of varicose veins. *Vasc Med* 2010; 15(1):27-32.
146. Navarro L, Min RJ, Bone C. Endovenous laser: a new minimally invasive method of treatment for varicose veins--preliminary observations using an 810 nm diode laser. *Dermatol Surg* 2001; 27(2):117-122.
147. Nemoto H, Mo M, Ito T, et al. Venous thromboembolism complications after endovenous laser ablation for varicose veins and role of duplex ultrasound scan. *Journal of Vascular Surgery* 2019; 7(6):817-823.
148. Nishibe T, Nishibe M, Suzuki S, et al. Venous hemodynamic improvement after endovenous radiofrequency ablation of saphenous varicose veins. *Int Angiol* 2017; 36(1):64-68.
149. Nordon IM, Hinchliffe RJ, Brar R, et al. A prospective double-blind randomized controlled trial of radiofrequency versus laser treatment of the great saphenous vein in patients with varicose veins. *Ann Surg* 2011; 254(6):876-881.
150. Novotny K, Rocek M, Padr R, et al. Treating great and small saphenous vein insufficiency with histoacryl in patients with symptomatic varicose veins and increased risk of surgery. *Vasa* 2018; 47(5):416-424.
151. Nwaejike N, Srodon PD, Kyriakides C. Endovenous laser ablation for the treatment of recurrent varicose vein disease--a single centre experience. *International Journal Of Surgery* 2010; 8(4):299-301.
152. O'Hare JL, Parkin D, Vandenbroeck CP, et al. Mid term results of ultrasound guided foam sclerotherapy for complicated and uncomplicated varicose veins. *European Journal of Vascular & Endovascular Surgery* 2008; 36(1):109-113.
153. Obi AT, Reames BN, Rook TJ, et al. Outcomes associated with ablation compared to combined ablation and transilluminated powered phlebectomy in the treatment of venous varicosities. *Phlebology* 2016; 31(9):618-624.
154. Ontas H, Yavuz T, Acar AN, et al. Comparison of ultrasound results following endovenous laser ablation and radiofrequency ablation in the treatment of varicose veins. *Annali Italiani di Chirurgia* 2019; 90:457-462.
155. Onwudike M, Abbas K, Thompson P, et al. Editor's Choice - Role of Compression After Radiofrequency Ablation of Varicose Veins: A Randomised Controlled Trial<sup></sup>. *European Journal of Vascular & Endovascular Surgery* 2020; 60(1):108-117.
156. Pannier F, Rabe E, Maurins U. First results with a new 1470-nm diode laser for endovenous ablation of incompetent saphenous veins. *Phlebology* 2009; 24(1):26-30.
157. Pannier F, Rabe E. Mid-term results following endovenous laser ablation (EVLA) of saphenous veins with a 980 nm diode laser. *International Angiology* 2008; 27(6):475-481.
158. Pannier F, Rabe E, Rits J, et al. Endovenous laser ablation of great saphenous veins using a 1470 nm diode laser and the radial fibre--follow-up after six months. *Phlebology* 2011; 26(1):35-39.
159. Park SJ, Yim SB, Cha DW, et al. Endovenous laser treatment of the small saphenous vein with a 980-nm diode laser: early results. *Dermatologic Surgery* 2008; 34(4):517-524; discussion 524.
160. Parsi K, Panozzo B, Bull A, et al. Deep vein sclerosis following sclerotherapy: Ultrasonic and d-dimer criteria. *Phlebology* 2020; 35(5):325-336.
161. Perkowski P, Ravi R, Gowda RC, et al. Endovenous laser ablation of the saphenous vein for treatment of venous insufficiency and varicose veins: early results from a large single-center experience. *Journal of Endovascular Therapy* 2004; 11(2):132-138.
162. Perrins S, Cha A, Qaqish R, et al. Clinical and anatomic outcomes of endovenous radiofrequency ablation performed on symptomatic small-diameter great saphenous veins. *J Vasc Surg Venous Lymphat Disord* 2013; 1(3):245-249.
163. Pleister I, Evans J, Vaccaro PS, et al. Natural history of the great saphenous vein stump following endovenous laser therapy. *Vascular & Endovascular Surgery* 2008; 42(4):348-351.
164. Poschinger-Figueiredo D, Virgini-Magalhaes CE, Porto LC, et al. Radiofrequency Ablation for Axial Reflux Associated with Foam Sclerotherapy for Varicosities in One-Step Approach: A Prospective Cohort Study Comprising Large Diameters Saphenous Veins. *Vasc Health Risk Manag* 2021; 17:379-387.
165. Proebstle TM, Vago B, Alm J, et al. Treatment of the incompetent great saphenous vein by endovenous radiofrequency powered segmental thermal ablation: first clinical experience. *J Vasc Surg* 2008; 47(1):151-156.
166. Proebstle TM, Gul D, Kargl A, et al. Endovenous laser treatment of the lesser saphenous vein with a 940-nm diode laser: early results. *Dermatol Surg* 2003; 29(4):357-361.
167. Proebstle TM, Moehler T, Herdemann S. Reduced recanalization rates of the great saphenous vein after endovenous laser treatment with increased energy dosing: definition of a threshold for the endovenous fluence equivalent. *J Vasc Surg* 2006; 44(4):834-839.
168. Puggioni A, Marks N, Hingorani A, et al. The safety of radiofrequency ablation of the great saphenous vein in patients with previous venous thrombosis. *J Vasc Surg* 2009; 49(5):1248-1255.
169. Rasmussen LH, Lawaetz M, Bjoern L, et al. Randomized clinical trial comparing endovenous laser ablation, radiofrequency ablation, foam sclerotherapy and surgical stripping for great saphenous varicose veins. *British Journal of Surgery* 2011; 98(8):1079-1087.
170. Rasmussen LH, Bjoern L, Lawaetz M, et al. Randomized trial comparing endovenous laser ablation of the great saphenous vein with high ligation and stripping in patients with varicose veins: short-term results. *J Vasc Surg* 2007; 46(2):308-315.
171. Rass K, Frings N, Glowacki P, et al. Comparable effectiveness of endovenous laser ablation and high ligation with stripping of the great saphenous vein: two-year results of a randomized clinical trial (RELACS study). *Arch Dermatol* 2012; 148(1):49-58.
172. Rathod J, Taori K, Joshi M, et al. Outcomes using a 1470-nm laser for symptomatic varicose veins. *Journal of Vascular & Interventional Radiology* 2010; 21(12):1835-1840.
173. Ravi R, Rodriguez-Lopez JA, Trayler EA, et al. Endovenous ablation of incompetent saphenous veins: a large single-center experience. *J Endovasc Ther* 2006; 13(2):244-248.
174. Reich-Schupke S, Doerler M, Altmeyer P, et al. Foam sclerotherapy with enoxaparin prophylaxis in high-risk patients with postthrombotic syndrome. *Vasa* 2013; 42(1):50-55.
175. Rustempasic N, Cvorak A, Agincic A. Outcome of endovenous laser ablation of varicose veins. *Acta Informatica Medica* 2014; 22(5):329-332.
176. Ryer EJ, Elmore JR, Garvin RP, et al. Value of delayed duplex ultrasound assessment after endothermal ablation of the great saphenous vein. *J Vasc Surg* 2016; 64(2):446-451 e1.
177. Samuel N, Wallace T, Carradice D, et al. Comparison of 12-w versus 14-w endovenous laser ablation in the treatment of great saphenous varicose veins: 5-year outcomes from a randomized controlled trial. *Vasc Endovascular Surg* 2013; 47(5):346-352.
178. Schwarz T, von Hodenberg E, Furtwangler C, et al. Endovenous laser ablation of varicose veins with the 1470-nm diode laser. *J Vasc Surg* 2010; 51(6):1474-1478.
179. Sermsathanasawadi N, Voravitvet TY, Chinsakchai K, et al. Risk factors for endovenous heat-induced thrombosis after endovenous radiofrequency ablation performed in Thailand. *Phlebology* 2016; 31(8):582-587.
180. Sevil F, Colak A, Jr., Ceviz M, et al. The Effectiveness of Endovenous Radiofrequency Ablation Application in Varicose Vein Diseases of the Lower Extremity. *Cureus* 2020; 12(4):e7640.
181. Shadid N, Ceulen R, Nelemans P, et al. Randomized clinical trial of ultrasound-guided foam sclerotherapy versus surgery for the incompetent great saphenous vein. *Br J Surg* 2012; 99(8):1062-1070.
182. Shoab SS, Lowry D, Tiwari A. Effect of treated length in endovenous laser ablation of great saphenous vein on early outcomes. *Journal of Vascular Surgery* 2016; 4(4):416-421.
183. Sharif MA, Soong CV, Lau LL, et al. Endovenous laser treatment for long saphenous vein incompetence. *Br J Surg* 2006; 93(7):831-835.
184. Shepherd AC, Gohel MS, Brown LC, et al. Randomized clinical trial of VNUS ClosureFAST radiofrequency ablation versus laser for varicose veins. *Br J Surg* 2010; 97(6):810-818.
185. Shutze WP, Kane K, Fisher T, et al. The effect of wavelength on endothermal heat-induced thrombosis incidence after endovenous laser ablation. *J Vasc Surg Venous Lymphat Disord* 2016; 4(1):36-43.
186. Smith PC. Chronic venous disease treated by ultrasound guided foam sclerotherapy. *Eur J Vasc Endovasc Surg* 2006; 32(5):577-583.
187. Spinedi L, Stricker H, Keo HH, et al. Feasibility and safety of flush endovenous laser ablation of the great saphenous vein up to the saphenofemoral junction. *Journal of Vascular Surgery* 2020; 8(6):1006-1013.
188. Spreafico G, Kabnick L, Berland TL, et al. Laser saphenous ablations in more than 1,000 limbs with long-term duplex examination follow-up. *Annals of Vascular Surgery* 2011; 25(1):71-78.
189. Starodubtsev V, Lukyanenko M, Karpenko A, et al. Endovenous laser ablation in patients with severe primary chronic venous insufficiency. *Int Angiol* 2017; 36(4):368-374.
190. Stone PAA, P. A.; Shames, M. L.; Back, M. R.; Johnson, B. L.; Flaherty, S. K.; Bandyk, B. F. Impact of Postoperative Duplex Surveillance after Radiofrequency Ablation of the Greater Saphenous Vein. *Journal for Vascular Ultrasound* 2006; 30(2):65-68.
191. Sufian S, Arnez A, Labropoulos N, et al. Endothermal venous ablation of the saphenous vein on patients who are on anticoagulation therapy. *International Angiology* 2017; 36(3):268-274.
192. Sufian S, Arnez A, Labropoulos N, et al. Incidence, progression, and risk factors for endovenous heat-induced thrombosis after radiofrequency ablation. *J Vasc Surg Venous Lymphat Disord* 2013; 1(2):159-164.
193. Sufian S, Arnez A, Labropoulos N, et al. Radiofrequency ablation of the great saphenous vein, comparing one versus two treatment cycles for the proximal vein segment. *Phlebology* 2015; 30(10):724-728.
194. Sutton PA, El-Dhuwaib Y, Dyer J, et al. The incidence of post operative venous thromboembolism in patients undergoing varicose vein surgery recorded in Hospital Episode Statistics. *Annals of the Royal College of Surgeons of England* 2012; 94(7):481-483.
195. Sydnor M, Mavropoulos J, Slobodnik N, et al. A randomized prospective long-term (>1 year) clinical trial comparing the efficacy and safety of radiofrequency ablation to 980 nm laser ablation of the great saphenous vein. *Phlebology* 2017; 32(6):415-424.
196. Takahashi K, Ito H, Katsube T, et al. Association between antithrombotic therapy and risk of postoperative complications among patients undergoing endovenous laser ablation. *Journal of Vascular Surgery* 2017; 5(3):339-345.
197. Tamura K, Maruyama T. Mid-Term Report on the Safety and Effectiveness of Endovenous Radiofrequency Ablation for Varicose Veins. *Ann Vasc Dis* 2017; 10(4):398-401.
198. Tang TY, Kam JW, Gaunt ME. ClariVein(R) - Early results from a large single-centre series of mechanochemical endovenous ablation for varicose veins. *Phlebology* 2017; 32(1):6-12.
199. Tawfik AM, Sorour WA, El-Laboudy ME. Laser ablation versus mechanochemical ablation in the treatment of primary varicose veins: A randomized clinical trial. *J Vasc Surg Venous Lymphat Disord* 2020; 8(2):211-215.
200. Theivacumar NS, Beale RJ, Mavor AI, et al. Initial experience in endovenous laser ablation (EVLA) of varicose veins due to small saphenous vein reflux. *European Journal of Vascular & Endovascular Surgery* 2007; 33(5):614-618.
201. Theivacumar NS, Gough MJ. Influence of warfarin on the success of endovenous laser ablation (EVLA) of the great saphenous vein (GSV). *Eur J Vasc Endovasc Surg* 2009; 38(4):506-510.
202. Timperman PE, Sichlau M, Ryu RK. Greater energy delivery improves treatment success of endovenous laser treatment of incompetent saphenous veins. *J Vasc Interv Radiol* 2004; 15(10):1061-1063.
203. Todd KL, 3rd, Wright DI, Group V-I. The VANISH-2 study: a randomized, blinded, multicenter study to evaluate the efficacy and safety of polidocanol endovenous microfoam 0.5% and 1.0% compared with placebo for the treatment of saphenofemoral junction incompetence. *Phlebology* 2014; 29(9):608-618.
204. Tolva VS, Cireni LV, Bianchi PG, et al. Radiofrequency ablation of the great saphenous vein with the ClosureFAST procedure: mid-term experience on 400 patients from a single centre. *Surg Today* 2013; 43(7):741-744.
205. Toniolo J, Chiang N, Munteanu D, et al. Vein diameter is a predictive factor for recanalization in treatment with ultrasound-guided foam sclerotherapy. *Journal of Vascular Surgery* 2018; 6(6):707-716.
206. Trip-Hoving M, Verheul JC, van Sterkenburg SM, et al. Endovenous laser therapy of the small saphenous vein: patient satisfaction and short-term results. *Photomedicine and Laser Surgery* 2009; 27(4):655-658.
207. Uthoff H, Holtz D, Broz P, et al. Rivaroxaban for thrombosis prophylaxis in endovenous laser ablation with and without phlebectomy. *J Vasc Surg Venous Lymphat Disord* 2017; 5(4):515-523.
208. Vahaaho S, Mahmoud O, Halmesmaki K, et al. Randomized clinical trial of mechanochemical and endovenous thermal ablation of great saphenous varicose veins. *Br J Surg* 2019; 106(5):548-554.
209. van Eekeren RR, Boersma D, Konijn V, et al. Postoperative pain and early quality of life after radiofrequency ablation and mechanochemical endovenous ablation of incompetent great saphenous veins. *J Vasc Surg* 2013; 57(2):445-450.
210. Vasquez MA, Wang J, Mahathanaruk M, et al. The utility of the Venous Clinical Severity Score in 682 limbs treated by radiofrequency saphenous vein ablation. *J Vasc Surg* 2007; 45(5):1008-1014; discussion 1015.
211. von Hodenberg E, Zerweck C, Knittel M, et al. Endovenous laser ablation of varicose veins with the 1470 nm diode laser using a radial fiber - 1-year follow-up. *Phlebology* 2015; 30(2):86-90.
212. Vuylsteke ME, Vandekerckhove PJ, De Bo T, et al. Use of a new endovenous laser device: results of the 1,500 nm laser. *Ann Vasc Surg* 2010; 24(2):205-211.
213. Vuylsteke M, De Bo TH, Dompe G, et al. Endovenous laser treatment: is there a clinical difference between using a 1500 nm and a 980 nm diode laser? A multicenter randomised clinical trial. *Int Angiol* 2011; 30(4):327-334.
214. Watanabe S, Okamura A, Iwamoto M, et al. A randomized controlled trial to evaluate the safety and efficacy of transluminal injection of foam sclerotherapy compared with ultrasound-guided foam sclerotherapy during endovenous catheter ablation in patients with varicose veins. *Journal of Vascular Surgery* 2021; 10:10.
215. Weiss RA, Weiss MA. Controlled radiofrequency endovenous occlusion using a unique radiofrequency catheter under duplex guidance to eliminate saphenous varicose vein reflux: a 2-year follow-up. *Dermatol Surg* 2002; 28(1):38-42.
216. Welch HJ. Endovenous ablation of the great saphenous vein may avert phlebectomy for branch varicose veins. *Journal of Vascular Surgery* 2006; 44(3):601-605.
217. Yamamoto K, Miwa S, Yamada T, et al. Strategy to prevent nerve injury and deep vein thrombosis in radiofrequency segmental thermal ablation of the saphenous veins using a new objective pain scale. *Phlebology* 2021; 36(8):659-664.
218. Yang GK, Parapini M, Gagnon J, et al. Comparison of cyanoacrylate embolization and radiofrequency ablation for the treatment of varicose veins. *Phlebology* 2019; 34(4):278-283.
219. Yilmaz S, Ceken K, Alparslan A, et al. Endovenous laser ablation and concomitant foam sclerotherapy: experience in 504 patients. *Cardiovascular & Interventional Radiology* 2012; 35(6):1403-1407.
220. Zhu Y, Wu D, Sun D, et al. Ultrasound- and fluoroscopy-guided foam sclerotherapy for lower extremity venous ulcers. *Journal of Vascular Surgery* 2020; 8(5):783-788.
221. Zuniga JM, Hingorani A, Ascher E, et al. Short-term outcome analysis of radiofrequency ablation using ClosurePlus vs ClosureFast catheters in the treatment of incompetent great saphenous vein. *Journal of Vascular Surgery* 2012; 55(4):1048-1051.
